# MUC1 Tissue Expression and Its Soluble Form CA15-3 Identify a Clear Cell Renal Cell Carcinoma with Distinct Metabolic Profile and Poor Clinical Outcome

**DOI:** 10.3390/ijms232213968

**Published:** 2022-11-12

**Authors:** Giuseppe Lucarelli, Monica Rutigliano, Davide Loizzo, Nicola Antonio di Meo, Francesco Lasorsa, Mauro Mastropasqua, Eugenio Maiorano, Cinzia Bizzoca, Leonardo Vincenti, Michele Battaglia, Pasquale Ditonno

**Affiliations:** 1 Urology, Andrology and Kidney Transplantation Unit - Department of Emergency and Organ Transplantation, University of Bari, 70124 Bari, Italy; 2 Pathology Unit—Department of Emergency and Organ Transplantation, University of Bari, 70124 Bari, Italy; 3 Division of General Surgery, Polyclinic Hospital, 70124 Bari, Italy

**Keywords:** renal cell carcinoma, metabolomics, MUC1, CA15-3, survival

## Abstract

An altered metabolism is involved in the development of clear cell renal carcinoma (ccRCC). MUC1 overexpression has been found to be associated with advanced disease and poor prognosis. In this study, we evaluated the metabolomic profile of human ccRCC, according to MUC1 expression, and integrated it with transcriptomic data. Moreover, we analyzed the role of MUC1 in sustaining ccRCC aggressiveness and the prognostic value of its soluble form CA15-3. Integrated metabolomic and transcriptomic analysis showed that MUC1-expressing ccRCC was characterized by metabolic reprogramming involving the glucose and lipid metabolism pathway. In addition, primary renal cancer cells treated with a small interfering RNA targeting MUC1 (siMUC1) migrated and proliferated at a slower rate than untreated cancer cells. After cisplatin treatment, the death rate of cancer cells treated with siMUC1 was significantly greater than that of untreated cells. Kaplan–Meier curves showed significant differences in CSS and PFS among groups of patients with high versus low levels of CA15-3. In a multivariate analysis, CA15-3 was an independent adverse prognostic factor for cancer-specific and progression-free survival. In conclusion, MUC1 expressing ccRCC is characterized by a particular metabolic reprogramming. The inhibition of MUC1 expression decreases cell motility and viability and improves cisplatin susceptibility, suggesting that this pathway can regulate de novo chemotherapy resistance in ccRCC.

## 1. Introduction

Renal cell carcinoma (RCC) represents around 3–5% of all cancers, and recent estimates have calculated that in 2022 in the United States, 79,000 new cases of kidney cancer (50,290 in men and 28,710 in women) will be diagnosed and about 13,920 people (8960 men and 4960 women) will die from this disease [1].

Although the pathogenesis of RCC is still not fully understood, recent evidence has confirmed that RCC is fundamentally a metabolic disease. In fact, many studies have showed that alterations in metabolic pathways are involved in the development of RCC, and that in this cancer many mutated genes have a fundamental role in controlling cell metabolic activities [2,3,4,5]. Furthermore, the recent re-evaluation of cancer as a disease of cell metabolism has led to the discovery of specific oncometabolites that play a key role in tumor growth and progression, especially in other urologic tumors such as prostate and bladder cancer [6,7,8,9]. The data of the cancer genome atlas (TCGA) research network have showed that a reprogrammed metabolism represents a major feature in clear cell RCC (ccRCC) [10,11], and as a result of the introduction of high-throughput technology, researchers have been able to characterize RCC at a molecular level, as well as identify biomarkers that allow for more accurate prognostic categorization [12,13]. In particular, a metabolic reprogramming involving the glycolytic and the pentose phosphate pathways [14] as well as a lipidomic signature characterized by significant accumulation of essential fatty acids and polyunsaturated fatty acids (PUFAs) have been described [15,16]. Consistent with these findings, an increased expression of fatty acid elongase 2 and 5 (ELOVL2 and ELOVL5) and stearoyl-CoA desaturase-1 (SCD1) was described in ccRCC. In addition, an impaired mitochondrial bioenergetics and oxidative phosphorylation was observed as a consequence of NDUFA4L2 overexpression, a regulatory protein that reduces mitochondrial oxygen consumption through the inhibition of Complex I [17].

Up to 30% of RCC cases are discovered at an advanced stage [18], and there are currently no molecular markers that can help guide therapy; therefore, the identification of novel diagnostic and prognostic indicators represents a critical issue in clinical management of this tumor [19,20,21,22,23,24,25]. 

Mucin 1 (MUC1) is a membrane-associated *O*-glycoprotein with an extended extracellular domain [26]. MUC1 serves as a sensor of extracellular signals and transmits these cues into the cell by its cytoplasmic domain. The reprogramming of the transcriptional profile allows MUC1 to regulate many biological activities. In cancer cells, MUC1 promotes cell growth, migration and invasion, and it is involved in metastatic progression [27]. Moreover, MUC1 undergoes aberrant glycosylation as result of an impaired glycosyltransferase activity. The carbohydrate side chains become short and truncated, and form new chains including Tn (GalNAc), TF (Gal(β1-3)GalNAc) and sialyl-Tn (STn) antigen (Neu5Ac(α2-6)GalNAc) [28]. Aberrant glycosylation exposes cryptic epitopes that can be identified by antibodies, and so these cancer-specific immunogenic regions represent good candidates for target therapy [29].

In addition, recent findings indicate that MUC1 acts as a metabolic regulator, controlling the expression of genes involved in biosynthetic pathways and modulating the activities of metabolic enzymes [30].

In ccRCC, MUC1 overexpression has been found to be associated with advanced RCC and poor prognosis [31,32,33,34]. However, the role of MUC1 in renal cancer remains unknown, especially in the context of metabolic reprogramming. 

In this study, we evaluated the metabolomic profile of human ccRCC according to MUC1 expression and integrated it with transcriptomic data to associate the alterations in cancer metabolism with gene expression changes. Moreover, we analyzed the role of MUC1 in sustaining ccRCC aggressiveness and the prognostic value of its soluble form CA15-3 in a cohort of patients diagnosed with kidney cancer.

## 2. Results

### 2.1. Global Metabolic Profile Distinguishes MUC1H from MUC1L ccRCC

**Figure 1:**
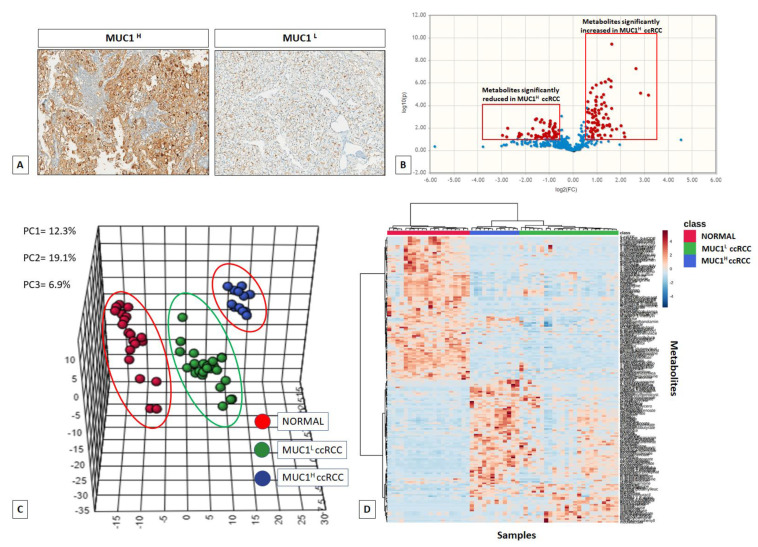
MUC1 expression in ccRCC specimens (**A**). Volcano plot of the 516 metabolites profiled; 116 exhibited significant differential abundance when comparing MUC1^H^ versus MUC1^L^ ccRCC (FC cut-off = 1.5 and *p* < 0.05) (**B**). Principal component analysis (PCA) of global tissue metabolome demonstrated that the three groups (MUC1^H^ tumors vs. MUC1^L^ tumors vs. normal renal tissues) were clearly distinguishable (**C**). Hierarchical clustering heatmap analysis of metabolites in normal, MUC1^H^ and MUC1^L^ cancer tissues (**D**).

MUC1 expression was evaluated by immunohistochemistry on normal and pathological tissues, stratifying samples according to staining intensity as tumor with high MUC1 expression (MUC1^H^) and tumor with low MUC1 expression (MUC1^L^) (Figure 1A).

Next, untargeted metabolomic analysis was performed on 56 kidney-derived tissues, including 20 normal tissues, 12 MUC1^H^ and 24 MUC1^L^ ccRCC (Supplementary Table S1), using LC-MS and GC-MS platforms. In total, 516 metabolites were identified, and 116 were found to be differentially expressed in MUC1^H^ compared to MUC1^L^ tumors (Figure 1B). The application of principal component analysis (PCA) to differentiate normal and pathological samples as a function of the global tissue metabolome showed that they cluster into three separate groups and that they can be metabolically differentiated (Figure 1C,D).

**Figure 2:**
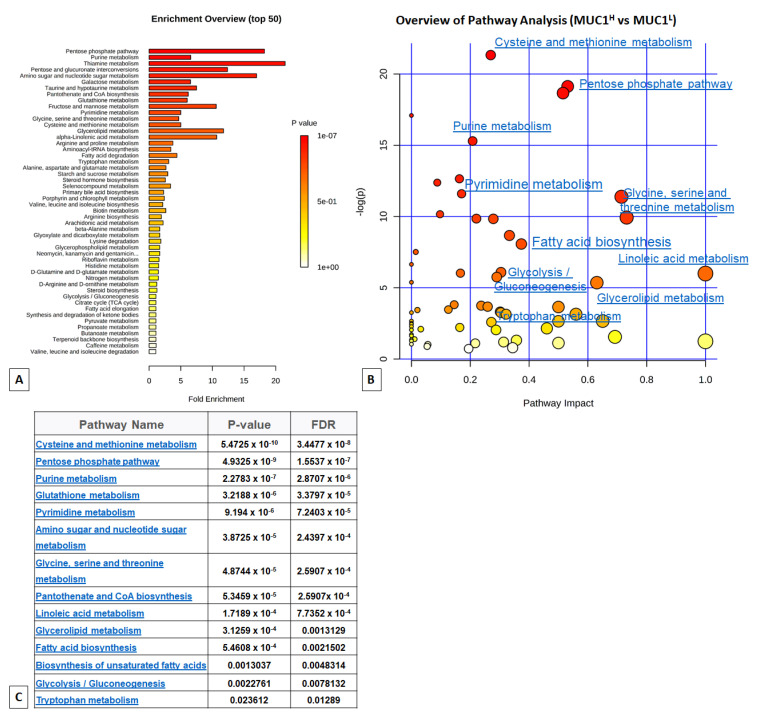
(**A**) Metabolic set enrichment analysis (MSEA) showing the most altered biochemical metabolic pathways in MUC1^H^ compared to MUC1^L^ ccRCC (Enrichment overview). (**B**) In the enrichment analysis map, all the matched pathways are displayed as circles (Pathway analysis). The color and size of each circle are based on the *p*-value and pathway impact value, respectively. (**C**) Results of pathway analysis are listed according to *p*-value and false discovery rate (FDR).

To obtain a global overview of altered biochemical processes, we performed a metabolite set enrichment analysis (MSEA) using MetaboAnalyst 4.0 [35]. This functional approach showed that alterations in glucose utilization, including PPP, one-carbon and lipid metabolism (linoleic acid and glycerolipids metabolism, and unsaturated fatty acid biosynthesis) had the highest impact in MUC1^H^ cancer cell metabolism compared to MUC1^L^ tumors (Figure 2).

### 2.2. MUC1 Expression Distinguishes ccRCC with Different Perturbations of Glucose Metabolism 

**Figure 3:**
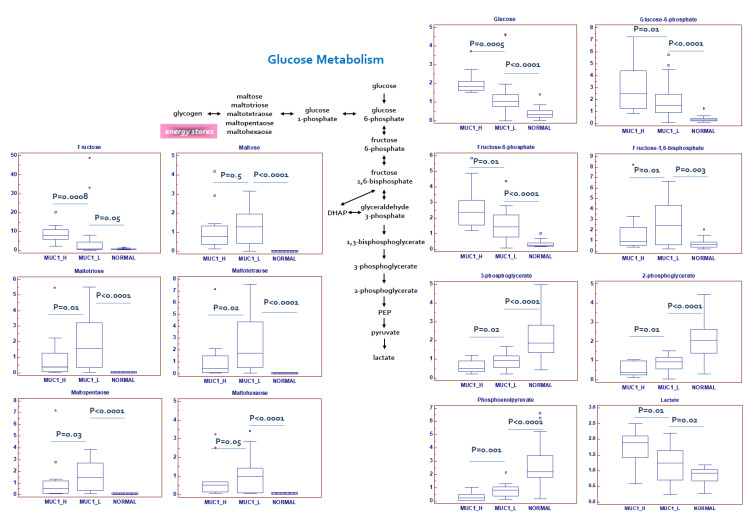
Schematic model summarizing the differences in glucose metabolism between normal, MUC1^H^ and MUC1^L^ tumor tissues. Y -axis: metabolite relative amount.

The Warburg effect is defined as the increase in the rate of glucose uptake and preferential production of lactate, even in the presence of oxygen. We previously showed that in ccRCC, oncogenic signaling pathways promote cancer through rerouting the sugar metabolism [14]. MUC1^H^ renal cancer cells showed a signature of an increased glucose uptake (glucose transporter GLUT1 was overexpressed in this subset compared to MUC1^L^ cells, Figure 1) and utilization, with glucose levels significantly elevated, along with higher levels of other sugars (such as fructose) and their phospho-derivatives (Figure 3).

**Figure 4:**
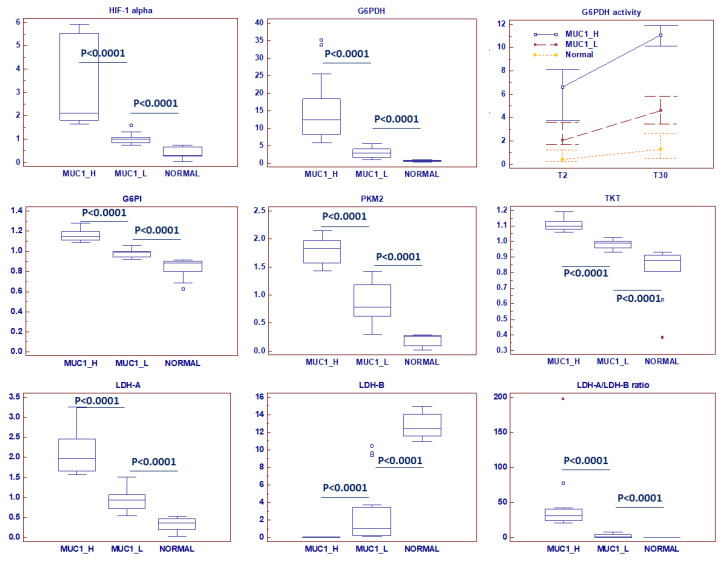
Protein expression analysis of HIF-1α (hypoxia-inducible factor 1-α), G6PDH (Glucose-6-phosphate dehydrogenase) and its enzymatic activity, G6PI (glucose-6-phosphate isomerase), PKM2 (pyruvate kinase isoform M2), TKT (transketolase), LDHA (L-lactate dehydrogenase A chain) and LDHB (L-lactate dehydrogenase B chain), in normal, MUC1^H^ and MUC1^L^ tumor tissues.

Glycogenolysis was partially inhibited in MUC1^H^ ccRCC, and significant reduction in glycogen degradation products were observed. MUC1^H^ compared to MUC1^L^ tumor and normal tissues showed increased glucose levels associated with elevations in upstream glycolytic intermediates (glucose 6-phosphate and fructose 6-phosphate), reductions in downstream intermediates (fructose 1,6-bisphosphate, 3-phosphoglycerate, 2-phosphoglycerate and phosphoenolpyruvate) and increased lactate production (MUC1^H^ vs. MUC1^L^ vs. normal) (Figure 3). Analysis of the glycolytic enzymes glucose-6-phosphate isomerase (G6PI), L-lactate dehydrogenase A chain (LDH-A) and pyruvate kinase isoform M2 (PKM2) demonstrated an increased expression of these proteins in MUC1^H^ ccRCC as compared with MUC1^L^ tumors and non-neoplastic tissue. Conversely, the expression of L-lactate dehydrogenase B chain (LDH-B) was significantly higher in normal kidney tissue, and the LDH-A/LDH-B ratio between the three groups was in accordance with the higher-efficiency lactate production observed in MUC1^H^ ccRCC (Figure 4). These results had an expression profile compatible with that obtained by real-time PCR, as shown in Supplementary Figure S2.

The increased lactate production found in MUC1^H^ ccRCC is likely related to the particular LDH isoform expressed in this tumor. LDH exhibits five isoforms assembled in tetramers of either of the two types of subunits, namely LDH-A and LDH-B. MUC1^H^ tumors showed a reduced expression of LDH-B and higher levels of LDH-A, thus increasing the levels of LDH-5 (LDH-A4) which is the most effective isoenzyme in producing lactate. 

Taken together, these findings are consistent with the observation of significantly higher hypoxia-inducible factor 1-α (HIF-1α) levels in MUC1H ccRCC (*p* < 0.0001; Figure 4), indicating that MUC1 and HIF-1α transcriptionally activate these metabolic genes. In fact, it has been shown that MUC1 physically occupies the promoter of glycolytic genes such as GLUT1 and LDHA, and also MUC1 interacts with HIF-1α, regulating its stability and activity [36].

This signature, characterized by the higher levels of glucose 6-phosphate and fructose 6-phosphate, associated with the increased tissue level of ribulose 5-phosphate, elevated levels of G6PDH and an increased activity of this enzyme (Figure 4), suggested an increased shunting of the metabolites of the upper part of glycolysis into the pentose phosphate pathway in MUC1^H^ tumors.

### 2.3. Alterations in Pentose Phosphate Pathway (PPP) Activity in MUC1^H^ Versus MUC1^L^ ccRCC 

**Figure 5:**
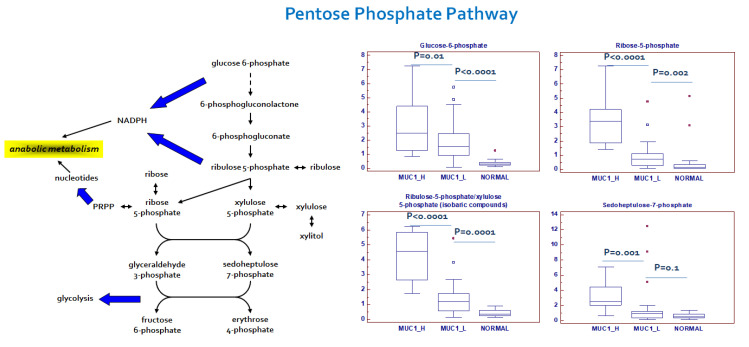
Schematic model summarizing the differences in pentose phosphate pathway between normal, MUC1^H^ and MUC1^L^ tumor tissues. Y-axis: metabolite relative amount.

In addition to the described alterations in glycolytic intermediates, metabolites related to PPP were also perturbed in the tumor samples. The PPP plays an important role in cancer cell biology, generating precursors for the nucleotides’ biosynthesis and producing NADPH for oxidoreductive and anabolic reactions. An increased PPP activity was observed mainly in MUC1^H^ ccRCC, as shown by higher levels of the intermediates sedoheptulose 7-phosphate, ribose 5-phosphate and ribulose 5-phosphate/xylulose 5-phosphate (isobaric compounds) (Figure 5). 

These findings, in association with the increased enzymatic activity of G6PDH and overexpression of transketolase (TKT), are suggestive of an increased activation of this pathway for anabolic reactions, especially in MUC1^H^ tumors (Figure 4). Taken together, these findings suggest that the metabolic flux through glycolysis is differentially partitioned, especially in MUC1^H^ ccRCC. In fact, while the sugars produced in the upper part of glycolysis are shifted to the PPP to promote anabolic reactions and redox homeostasis, the triose phosphates generated in the lower part are diverted towards the TCA cycle and one-carbon metabolism.

### 2.4. Changes in Tricarboxylic Acid (TCA) Cycle Intermediates and Related Metabolites 

**Figure 6:**
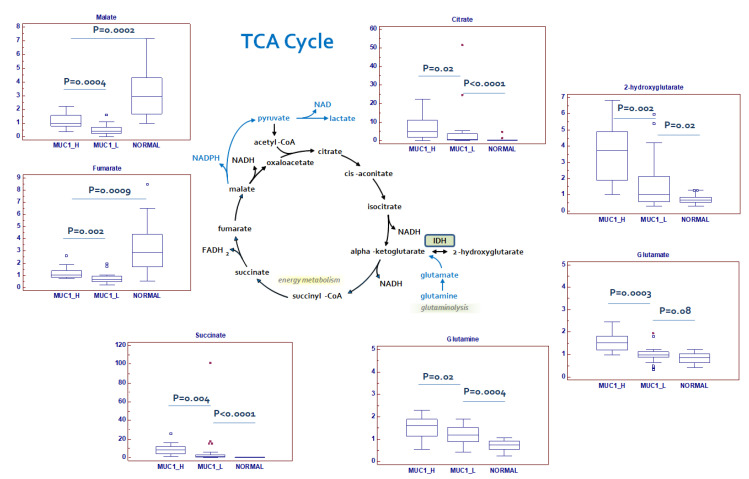
Schematic model summarizing the differences in tricarboxylic acid (TCA) cycle metabolites between normal, MUC1^H^ and MUC1^L^ tumor tissues. Y-axis: metabolite relative amount.

In MUC1^H^ tumors, citrate and succinate were markedly increased when compared to MUC1^L^ ccRCC and normal tissue, whereas fumarate and malate were significantly reduced in cancer tissue (Figure 6). 

**Figure 7:**
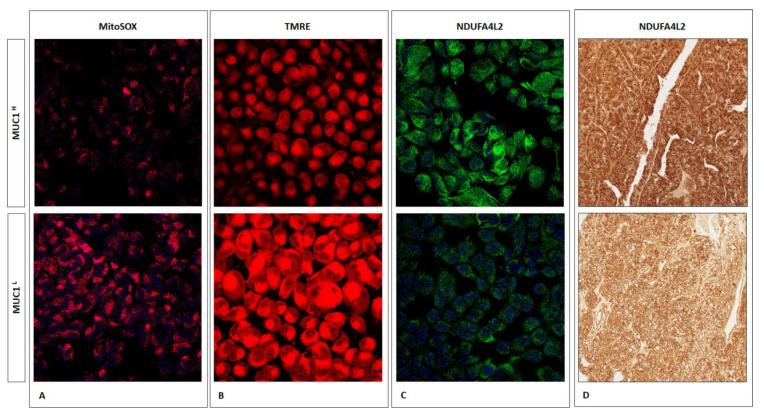
Immunofluorescence showing the increase in MitoSOX (superoxide radical production) (**A**) and TMRE (membrane potential) signal levels (**B**) in MUC1^L^ ccRCC compared to MUC1^H^ tumor. NDUFA4L is increased in MUC1^H^ ccRCC cells (**C**) and tissue (**D**).

The altered levels of TCA cycle intermediates, in association with a reduced reactive oxygen species (ROS) production (Figure 7A) and a decreased membrane potential (Figure 7B), suggests that the rate of oxidative phosphorylation is impaired in MUC1^H^ tumors, which is consistent with NDUFA4L2 overexpression observed MUC1^H^ samples (Figure 7C,D) [17].

The observed changes in the levels of Krebs cycle metabolites are suggestive of a metabolic signature that may be consistent with an increased glutaminolysis. In fact, nearly all the amino acids were significantly reduced in tumor tissue, with the exception of glutamine, glutamate and cysteine, which were significantly elevated in MUC1^H^ tumors. In this scenario it has been shown that cancer cells with defective mitochondria use glutamine-dependent reductive carboxylation rather than oxidative metabolism as the major pathway of citrate formation [37].

### 2.5. Alterations in Redox Homeostasis

**Figure 8:**
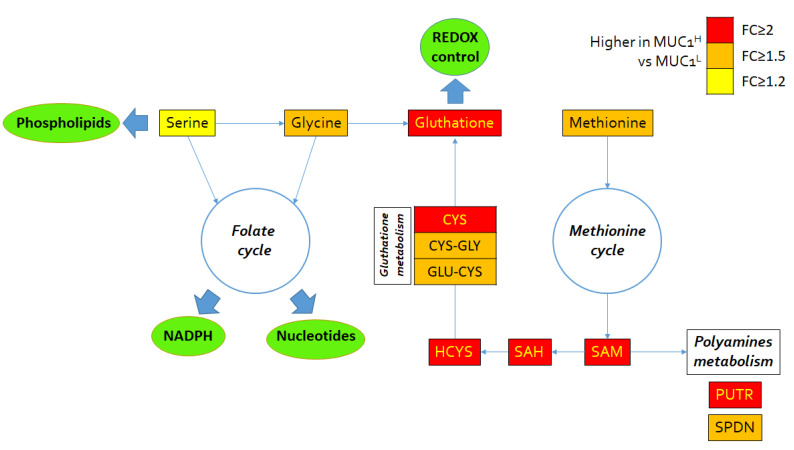
Schematic model summarizing the differences in glutathione metabolism, folate and methionine cycle metabolites between MUC1^H^ and MUC1^L^ tumor tissues. FC: fold change.

Cancer cells are exposed to high amounts of oxidative stress as the result of increased metabolic activity. In MUC1^H^ tumor samples, the accumulation of several powerful antioxidants, including alpha-tocopherol (*p* < 0.0001), beta-tocopherol (*p* < 0.0001), gamma-tocopherol (*p* < 0.0001), ascorbate (vitamin C) (*p* = 0.0005) and ergothioneine (*p* < 0.0001), was observed. The levels of the powerful cellular antioxidant glutathione (*p* = 0.01) were increased as well. Increased levels of glutathione precursors and maintenance of cysteine levels despite significant reductions in nearly all the other amino acids (except glutamine and glutamate) are suggestive of an increased glutathione synthesis in an attempt to maintain redox homeostasis (Figure 8). Conversely, the levels of the glutathione degradation product 5-oxoproline were significantly reduced in MUC1^H^ cancer tissues (*p* < 0.001). Moreover, the polyamines putrescine and spermidine, which bind to and stabilize DNA to promote cellular proliferation, were significantly elevated in MUC1^H^ versus MUC1^L^ tumor tissues (Figure 8).

### 2.6. Changes in Amino Acids and Lipid Metabolism 

**Figure 9:**
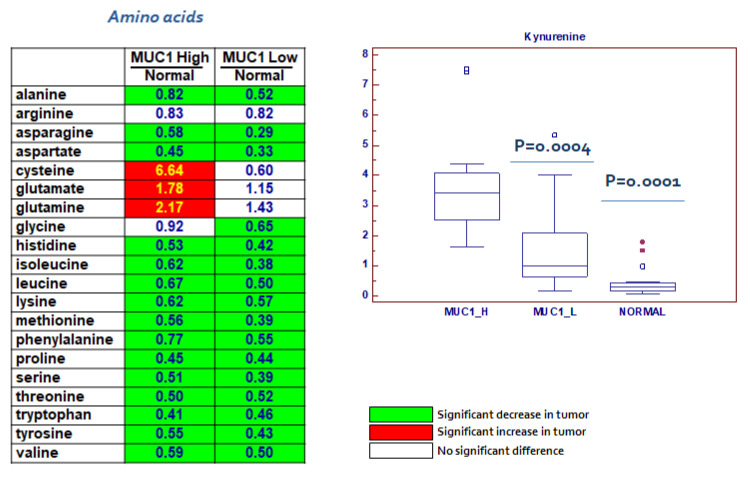
Alterations in amino acid metabolism.

In addition to the alteration in the biochemical pathways described above, modifications in amino acid metabolism were prevalent in MUC1^H^ cancer tissue, as evidenced by significant reductions in nearly all the free amino acids measured with the exception of cysteine, glutamate and glutamine (Figure 9). Taken together, these changes are indicative of a dynamic remodeling that includes protein degradation and the utilization of amino acids for different metabolic activities in cancer cells. Moreover, kynurenine, a metabolite of the amino acid tryptophan [38], was elevated in MUC1^H^ ccRCC compared to MUC1L tumors (*p* = 0.0004; Figure 9).

**Figure 10:**
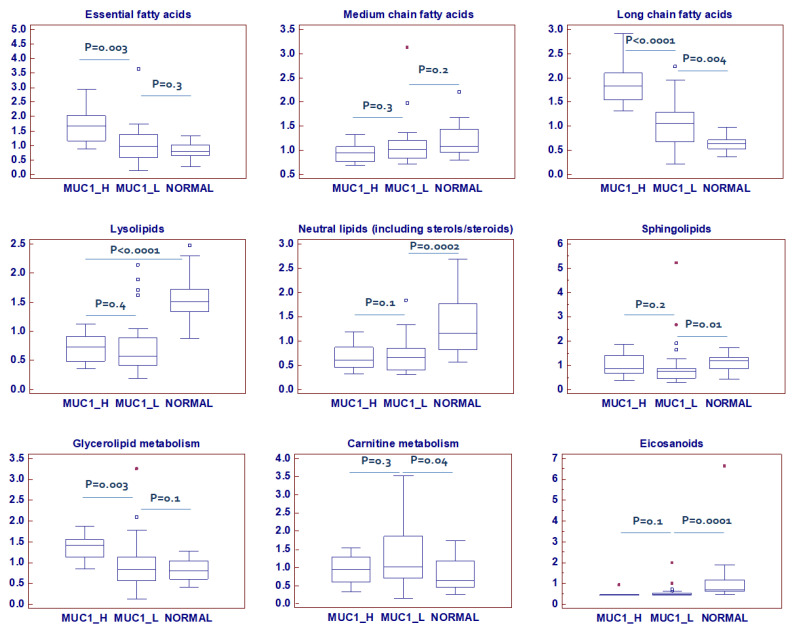
Lipid classes differentially accumulated between normal, MUC1^H^ and MUC1^L^ tumor tissues. Y-axis: metabolite relative amount.

Alteration of lipid metabolism represents another fundamental feature of ccRCC [15,39]. In a recent study [16], we showed that ccRCC was characterized by increased levels of very long-chain and polyunsaturated fatty acids and that this accumulation was sustained by overexpression of stearoyl-CoA desaturase and fatty acid elongase 2 and 5. Alterations in nearly all the free fatty acids measured were observed, and an accumulation of many essential and long-chain fatty acids was found in MUC1^H^ samples (Figure 10). These changes may be associated with an increased fatty acid uptake and/or synthesis for subsequent membrane biosynthesis and cell signaling to promote cellular growth and proliferation. Additional information on metabolites evaluated in each lipid class has been provided in the Supplementary Table S2.

### 2.7. Integrated Metabolomics/Transcriptomic Signature

To compare the relative changes in gene expression and metabolite abundance in MUC1^H^ versus MUC1^L^ ccRCC, we integrated the metabolomics data with gene expression data from the Jones cohort (GSE15641) including 23 normal kidney samples and 32 ccRCC samples, stratifying the patients according to MUC1 expression. The combined analysis identified 16 significantly enriched biochemical pathways (*p* < 0.05), including those of glycolysis, PPP, glutaminolysis and unsaturated fatty acid metabolism (Supplementary Figure S3). 

**Figure 11:**
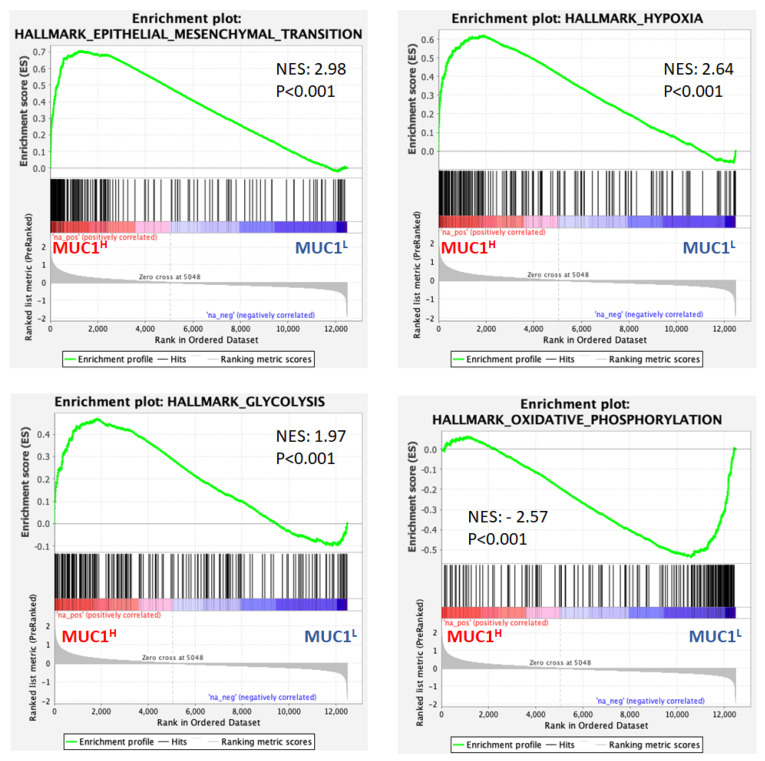
Gene set enrichment analysis (GSEA) of the GSE15641 dataset. NES: normalized enrichment score.

Moreover, gene set enrichment analysis (GSEA) [40] of the GSE15641 dataset showed that MUC1^H^ ccRCC compared to MUC1^L^ tumors featured multiple enriched gene sets depicting epithelial–mesenchymal transition, hypoxia, glycolysis and oxidative phosphorylation (Figure 11).

### 2.8. MUC1 Expression Is Associated with Increased Cancer Cell Proliferation and Migration and Has a Role in Chemotherapy Resistance

MUC1 has been implicated in various aspects of tumor biology including cell proliferation, migration and cancer drug resistance [41,42,43]. Therefore, to study the role of MUC1 in these processes, in vitro assays were performed. 

**Figure 12:**
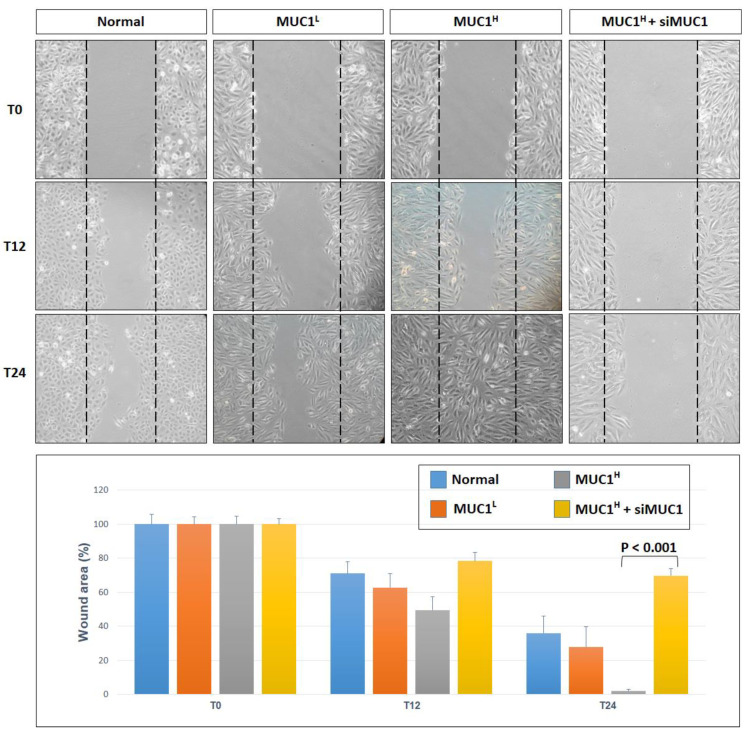
Wounded normal, MUC1^L^ and MUC1^H^ cancer cell monolayers were photographed 12 and 24 h after the mechanical scratch and the area of the wounds was measured in 3 independent wound sites per group. When specified, the cells were treated with small interfering RNA targeting MUC1 (siMUC1). MUC1^H^ RCC cells treated with siMUC1 have decreased cell migratory capabilities compared with untreated tumor cells.

The scratch wound-healing assay showed that primary MUC1^H^ ccRCC cells treated with siMUC1 had a decreased migratory ability compared with MUC1^L^ cancer cells (Figure 12).

**Figure 13:**
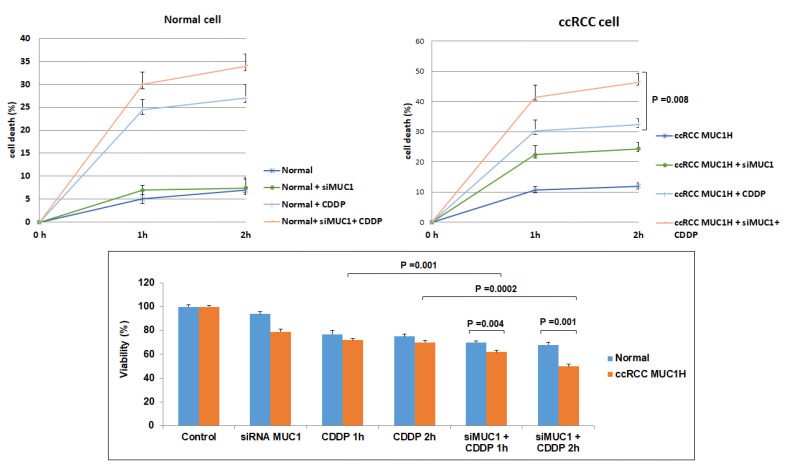
MUC1 has a role in ccRCC resistance to cisplatin (CDDP)-induced cytotoxicity. The death rate of treated MUC1^H^ tumor cells (MUC1^H^ + siMUC1 + CDDP) is significantly higher than that of untreated MUC1H cells (MUC1^H^ + CDDP) (*p* = 0.008). No difference is observed in normal cells. MTT assay reveals significantly decreased cell viability when MUC1^H^ RCC cells are treated with siMUC1 before cisplatin incubation.

In addition, MUC1-silenced renal cancer cells proliferated at a slower rate than non-silenced cancer cells. After cisplatin treatment, the death rate of tumor cells treated with siMUC1 was significantly greater than that of untreated cancer cells (*p* = 0.008, Figure 13). The MTT assay confirmed these findings, demonstrating a decreased cell viability when tumor cells were pre-treated with siMUC1 before cisplatin incubation (Figure 13).

### 2.9. MUC1 Soluble Form (Serum CA15-3) Is a Risk Factor for ccRCC Progression and Mortality

**Figure 14:**
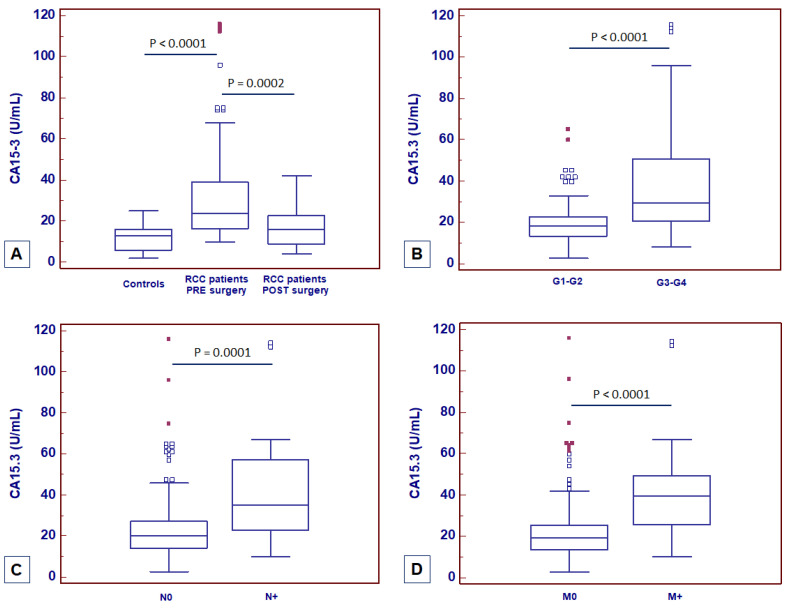
CA15-3 serum levels at baseline in patients with ccRCC before and after surgery (**A**) and stratified according to nuclear grade (**B**), lymph node involvement (**C**) and visceral metastases (**D**).

MUC1 soluble form, also known as CA15-3, is widely used as a serum marker for breast cancer [44]. Spearman’s test showed a positive correlation between MUC1 tissue expression and CA15-3 serum levels (rs = 0.63; *p* = 0.001). To evaluate its diagnostic and prognostic role in ccRCC, serum CA15-3 (normal range: 0–25 U/mL) was preoperatively measured in a cohort of 428 consecutive patients who underwent radical or partial nephrectomy for ccRCC at our institution and in 140 healthy adult volunteers with no evidence of malignancy. At the time of nephrectomy, CA15-3 serum levels were significantly higher in patients with ccRCC as compared with non-neoplastic patients (*p* < 0.0001; Figure 14A). One month after surgery, CA15-3 serum levels were significantly reduced (*p* = 0.0002; Figure 14A). Moreover, at the time of diagnosis significantly higher CA15-3 serum levels were observed in patients with higher nuclear grade (G3–4 vs. G1–2, *p* < 0.0001; Figure 14B), with lymph node involvement (N1 vs. N0, *p* = 0.0001; Figure 14C) and with visceral metastases (M1 vs. M0, *p* < 0.0001; Figure 14D).

**Figure 15:**
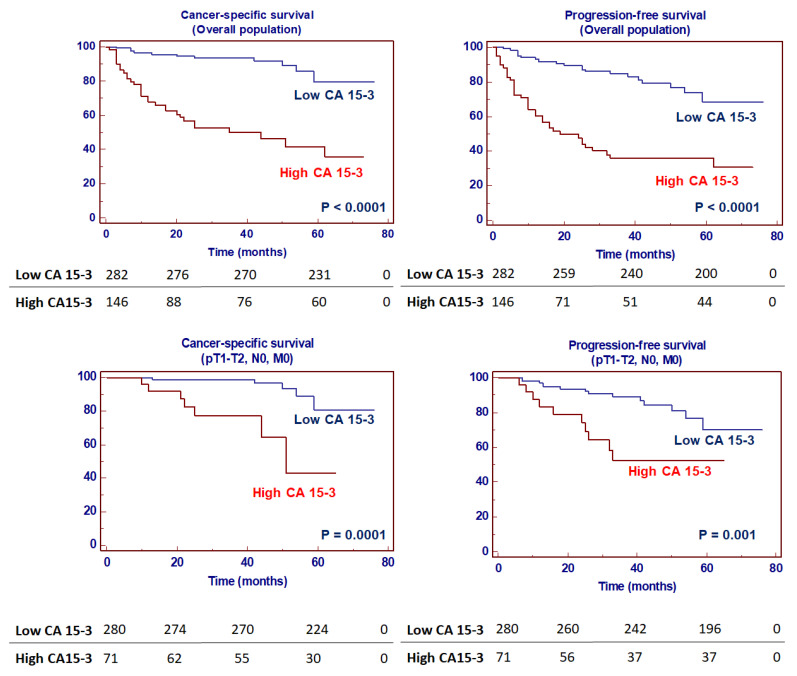
Kaplan–Meier cancer-specific and progression-free survival curves, stratified by serum CA15-3 levels in the overall population and in a subset of patients with localized disease.

Kaplan–Meier survival curves for cancer-specific survival (CSS) and progression-free survival (PFS), stratified by CA15-3 serum levels, are shown in Figure 15. Both CSS and PFS were significantly decreased in patients with high serum levels of CA15-3. In addition, subgroup analyses in localized (pT1-2, N0/M0) disease confirmed the prognostic value of CA15-3 in this subset of patients (Figure 15).

Univariate analysis for the predefined variables showed that the pathological stage, presence of nodal and visceral metastases, nuclear grade, presence of necrosis, tumor size, and high levels of CA15-3 were significantly associated with the risk of death (Table 1) and progression (Table 2). In a multivariate analysis by Cox regression modeling, the pathological stage, presence of nodal and visceral metastases, nuclear grade and increased CA15-3 serum level were independent adverse prognostic factors for CSS and PFS (Table 1 and Table 2).

## 3. Discussion

In this study, we delineated the metabolic alterations that characterize MUC1-expressing ccRCC using a combination of chromatography-coupled tandem mass spectrometry, bioinformatics and in vitro cell-based assays. MUC1 is a transmembrane glycoprotein associated with an aggressive phenotype in different epithelial cancers [26,27]. In addition to its role in regulating tumor cell survival, invasion and migration, recent findings indicate that MUC1 induces transcriptional modifications that result in metabolic reprogramming. Shukla et al. showed that MUC1 and HIF-1α crosstalk mediated an anabolic glucose metabolism reprogramming that increased pyrimidine pools in pancreatic cancer [43]. The increased nucleotide biosynthesis led to accumulation of deoxycytidine and reduced the effectiveness of gemcitabine by competitive inhibition.

Moreover, Gunda et al. demonstrated that MUC1-associated metabolic alterations had a role in reducing radiation-induced cytotoxicity and DNA damage in pancreatic cancer, by increasing PPP and nucleotide biosynthesis [45].

MUC1 is also aberrantly expressed in triple-negative breast cancer, where it contributes to epigenetic reprogramming, chromatin remodeling and chemoresistance [46]. In addition, MUC1 regulates glutamine metabolism, enhancing its uptake and carbon flux to glutaminolysis, amino acid metabolism and the TCA cycle [47].

In ccRCC, MUC1 expression is induced by the HIF/hypoxia pathway and promotes the migration and invasion of cancer cells [34]; however, the metabolic reprogramming associated with its overexpression is poorly understood.

In our study, the analysis of metabolic alterations and integration with transcriptomic data showed that MUC1^H^ ccRCC is characterized by metabolic alterations involving glucose and the lipid metabolism pathway. In particular, we found that in MUC1^H^ tumors, high glucose levels were accompanied by an increase in the upstream glycolytic intermediates and a decrease in the downstream metabolites. These findings in association with high expression levels of G6PDH, TKT and PPP intermediates suggest an increased rerouting of the sugar metabolism toward this pathway, with the aim of promoting both anabolic reactions and redox homeostasis, especially in MUC1^H^ ccRCC.

Alteration of lipid metabolism represents another important hallmark of ccRCC. In addition to their role in membrane formation (phospholipids, glycolipids and sterols), lipids represent a fundamental energetic substrate and produce second messengers through their phospholipase-dependent hydrolysis. The production of many bioactive second messengers, such as phosphatidic acid, diacylglycerol and arachidonic acid, can trigger the activation of several signaling pathways that promote cancer cell proliferation, survival, migration, invasion and metastatization [48,49]. 

It has been shown that ccRCC is characterized by a significant accumulation of polyunsaturated fatty acids (PUFAs), in association with overexpression of stearoyl-CoA desaturase-1 (Δ-9-desaturase; SCD1) and fatty acid elongase 2 and 5 (ELOVL2 and ELOVL5) [16]. In the present study, we found that these perturbations were more evident in MUC1^H^ ccRCC compared to MUC1^L^ tumors and normal tissues. In addition, MUC1^H^ tumors were characterized by accumulation of several powerful antioxidants, including glutathione, in association with increased expression of NDUFA4L2 and reduced mitochondrial membrane potential and ROS production, as shown by reduced signals of the fluorescence probe TMRE and MitoSOX. 

Overexpression of MUC1 provided additional advantages in different biological processes such as cell proliferation, cancer cell migration and resistance to chemotherapy.

RCC is typically a chemoresistant tumor, and although numerous mechanisms of chemoresistance have been described, the overexpression of P-glycoprotein is one of the most significant [50,51,52]. In addition to approaches targeting P-glycoprotein, other tactics to overcome drug resistance include MUC-1 targeting approaches. 

In accordance with previous studies performed in different tumors, we found that MUC1 knockdown decreased cell migration and viability and improved cisplatin susceptibility, suggesting that this protein can be involved in de novo chemotherapy resistance in ccRCC. 

Finally, we analyzed the prognostic role of CA15-3, a serum marker derived from MUC1 and widely used for breast cancer clinical management [44]. 

Previous studies suggested that high serum levels of CA15-3 could be associated with an advanced disease in RCC patients. Grankvist et al. [53] observed increased levels of CA15-3 in up to 30% of patients with high-grade and high-stage RCC. In a later study, Briasoulis et al. identified abnormal levels of this serum biomarker in 23% of RCC patients with metastatic disease [54]. In accordance with these results, in a more recent study we showed that CSS and PFS were significantly shorter for clear cell and non-clear cell RCC patients with elevated CA15-3 levels [20].

In the present study, multivariate analysis showed that high levels of CA15-3, together with the presence of nodal and visceral metastases, advanced stage and nuclear grade, were significantly predictive of risk of death. Moreover, this biomarker remained an independent prognosticator of outcome for PFS. Kaplan–Meier curves showed clear differences in CSS and PFS between patients with CA15-3 values below and above the normal values. Moreover, subgroup analyses for localized disease confirmed significant differences in CSS and PFS for CA15-3 values ≤ 25 or >25 U/mL in this clinical setting.

In conclusion, this study suggests that MUC1 acts as a metabolic regulator in ccRCC, inducing a particular metabolic reprogramming. The inhibition of MUC1 expression decreases cell motility and viability and improves cisplatin susceptibility, suggesting that this pathway can regulate de novo chemotherapy resistance in ccRCC. Finally, we showed that CA15-3 could serve as a promising biomarker to identify ccRCC patients with poor prognosis.

## 4. Materials and Methods

### 4.1. Study Population and Tissue Collection

Investigation has been conducted in accordance with the ethical standards and according to the Declaration of Helsinki and according to national and international guidelines and has been approved by the authors’ institutional review board. Written informed consent to take part was given by all participants.

Primary renal tumor (*n* = 36) and non-neoplastic tissues (*n* = 20) were collected from patients who underwent radical or partial nephrectomy for ccRCC (Supplementary Table S1). All specimens were immediately used to obtain primary cell cultures after frozen-section confirmation of the diagnosis. Two pathologists confirmed the presence of clear cell RCC in the neoplastic tissues and excluded tumor cells in the healthy specimens. Patients with diabetes mellitus and/or an estimated glomerular filtration rate (MDRD equation) < 60 mL/min/1.73 m^2^ were excluded from the study. 

For CA15-3 level evaluation, serum CA15-3 (normal range: 0–25 U/mL) was preoperatively measured in a cohort of 428 consecutive patients who underwent radical or partial nephrectomy for ccRCC at our institution and in 140 healthy adult volunteers with no evidence of malignancy. Patients with an estimated glomerular filtration rate (eGFR calculated using MDRD equation) <60 mL/min/1.73 m^2^ and with other known causes of elevated levels of this biomarker were excluded from the study (namely, patients with other malignant tumors, breast and liver diseases, endometriosis, sarcoidosis, tuberculosis and systemic lupus erythematosus). Detailed clinical and pathological characteristics of these patients are summarized in Supplementary Table S3. All patients were preoperatively staged by thoraco-abdominal Computed Tomography or Magnetic Resonance Imaging. Tumor staging was reassigned according to the seventh edition of the AJCC-UICC TNM classification. The 2016 World Health Organization classification was used to attribute histological type and nuclear grade. 

### 4.2. Metabolite Analysis

#### 4.2.1. Data Quality: Instrument and Process Variability

Metabolic profiling of RCC samples was carried out at Metabolon Inc. Instrument variability was determined by calculating the median relative standard deviation (RSD) for the internal standards that were added to each sample prior to injection in the mass spectrometers. Overall process variability was determined by calculating the median RSD for all endogenous metabolites (i.e., non-instrument standards) present in 100% of the Client Matrix samples, which are technical replicates of pooled client samples.

#### 4.2.2. Sample Preparation

All tissue samples were maintained at −80 °C until processed. At the time of analysis, samples were thawed, and extracts prepared according to Metabolon’s standard protocol, which is designed to remove protein, dislodge small molecules bound to protein or physically trapped in the precipitated protein matrix and recover a wide range of chemically diverse metabolites.

In particular, the sample preparation process was carried out using the Hamilton Company automated MicroLab STAR^®^ system. Recovery standards were added prior to the first step in the extraction process for QC purposes. Sample preparation was conducted using a proprietary series of organic and aqueous extractions to remove the protein fraction, while allowing maximum recovery of small molecules. The resulting extract was divided into two fractions: one for analysis by LC and one for analysis by GC. Samples were placed briefly on a TurboVap^®^ (Zymark, Hopkinton, MA, USA) to remove the organic solvent. Each sample was then frozen and dried under vacuum. Samples were then prepared for the appropriate instrument, either LC/MS or GC/MS. For QA/QC purposes, a number of additional samples were included in each day’s analyses. Furthermore, a selection of QC compounds was added to every sample, including those under test. These compounds were carefully chosen so as not to interfere with the measurement of the endogenous compounds. The Supplementary Tables S4 and S5 describe the QC samples and compounds. These QC samples were primarily used to evaluate the control process for each study as well as assisting in data curation.

#### 4.2.3. Liquid Chromatography/Mass Spectrometry (LC/MS, LC/MS)

The LC/MS portion of the platform was based on a Waters ACQUITY UPLC and a Thermo-Finnigan LTQ mass spectrometer, which consisted of an electrospray ionization (ESI) source and linear ion-trap (LIT) mass analyzer. The sample extract was split into two aliquots, dried, then reconstituted in acidic or basic LC-compatible solvents, each of which contained 11 or more injection standards at fixed concentrations. One aliquot was analyzed using acidic positive ion optimized conditions and the other using basic negative ion optimized conditions in two independent injections using separate dedicated columns. Extracts reconstituted in acidic conditions were gradient-eluted using water and methanol, both containing 0.1% formic acid, while the basic extracts, also reconstituted using water/methanol, contained 6.5 mM ammonium bicarbonate. The MS analysis alternated between MS and data-dependent MS2 scans using dynamic exclusion.

#### 4.2.4. Gas Chromatography/Mass Spectrometry (GC/MS)

The samples destined for GC/MS analysis were re-dried under vacuum desiccation for a minimum of 24 h prior to being derivatized under dried nitrogen using bistrimethyl-silyl-trifluoroacetamide (BSTFA). The GC column was 5% phenyl and the temperature ramped from 40 to 300 °C in a 16 min period. Samples were analyzed on a Thermo-Finnigan Trace DSQ fast-scanning single-quadrupole mass spectrometer using electron impact ionization. The instrument was tuned and calibrated for mass resolution and mass accuracy on a daily basis. The information output from the raw data files was automatically extracted as discussed below.

#### 4.2.5. Accurate Mass Determination and MS/MS Fragmentation (LC/MS, LC/MS/MS)

The LC/MS portion of the platform was based on a Waters ACQUITY UPLC and a Thermo-Finnigan LTQ-FT mass spectrometer, which had a linear ion-trap (LIT) front end and a Fourier transform ion cyclotron resonance (FT-ICR) mass spectrometer backend. For ions with counts exceeding 2 million, an accurate mass measurement could be performed. Accurate mass measurements could be made on the parent ion as well as fragments. The typical mass error was less than 5 ppm. Characterizing ions with less than two million counts is a more laborious process. Fragmentation spectra (MS/MS) were typically generated in a data-dependent manner, but if necessary, targeted MS/MS could be employed, as in the case of lower level signals.

#### 4.2.6. Compound Identification 

Compounds were identified by comparison to library entries of purified standards or recurrent unknown entities. The identification of known chemical entities was based on comparison to metabolomic library entries of purified standards.

### 4.3. Bioinformatics and Statistical Analyses

Significance tests were performed with MedCalc 9.2.0.1 (MedCalc software, Mariakerke, Belgium) and “R” (http://cran.r-project.org (accessed on 1 March 2022)). Global biochemical profiles were determined in human kidney tissue/tumor samples and compared across groups stratified by renal tissue pathology status. An estimate of the false discovery rate (*q*-value) was calculated to consider the multiple comparisons that normally occur in metabolomic-based studies. Comparisons of metabolite median values between different groups were evaluated by Mann–Whitney U test. Spearman’s correlation was applied to evaluate the association between MUC1 tissue expression and CA15-3 serum levels. In the cancer-specific survival (CSS) analysis, patients still alive or lost to follow-up were censored, as well as patients who died of RCC-unrelated causes. Progression-free survival (PFS) was calculated from the date of surgery to the date of disease recurrence. Disease progression was assessed radiographically (using CT scan or MRI) with a surveillance schedule based on EAU guidelines. Estimates of CSS and PFS were calculated according to the Kaplan–Meier method and compared with the log-rank test. Univariate and multivariate analyses were performed using the Cox proportional hazards regression model to identify the most significant variables for predicting CSS and PFS. A backward selection procedure was performed with removal criterion *p* > 0.10 based on likelihood ratio tests. A *p*-value of < 0.05 was considered statistically significant.

### 4.4. Integration of Metabolomic and Transcriptomic Data

To identify genes associated with high versus low MUC1 expression, gene expression data from the Jones cohort (GSE15641) including 23 normal kidney samples and 32 ccRCC samples were used. We first stratified the patients by MUC1 expression, and next we generated a rank file for each expressed gene by the log2 fold change in high MUC1 samples over low MUC1 samples. Next, we ran a gene set enrichment analysis (GSEA) to determine which pathways were statistically enriched across the renal cancer datasets [40]. The normalized enrichment score (NES) was used to evaluate the extent and direction of enrichment of each pathway.

MetaboAnalyst 4.0 (https://www.metaboanalyst.ca (accessed on 1 March 2022)) was used for the metabolite set enrichment (MSEA) and to integrate the data from the transcriptomics and metabolomics experiments [35]. The main function of this module is to pinpoint the pathways involved in the underlying biological processes by combining the evidence based on alterations in both gene expression and metabolite concentrations. In addition, a topology analysis was used to evaluate the relative importance of the gene/compounds based on their relative locations within a pathway. Over-representation analysis of the pathways was based on the hypergeometric test.

### 4.5. Real-Time PCR

Total RNA of normal and tumor tissues was reverse transcribed with the High-Capacity cDNA Reverse Transcription Kit (Applied Biosystems Foster City, CA, USA), following the manufacturer’s instructions. Quantitative real-time polymerase chain reactions (PCR) were performed using the iQTM SYBR Green Supermix buffer (6mMMgCl2, dNTPs, iTaq DNA polymerase, SYBR Green I, fluorescein and stabilizers) (BIO-RAD Laboratories, Hercules, CA, USA). The primers used for rea-time PCR are listed in Supplementary Table S6.

Quantification of the mRNA levels was performed on a MiniOpticon Real-Time PCR detection system (BIO-RAD Laboratories). In the PCR reactions, the following protocol was used: polymerase activation at 95 °C for 3 min, followed by 45 cycles at 95 °C for 10 s, 60 °C for 30 s. Melting curves were generated through 60 additional cycles (65 °C for 5 s with an increment of 0.5 °C/cycle). Gene expression results were obtained as mean Ct (threshold cycle) values of triplicate samples. Expression was determined using the 2-ΔΔCt method. Expression values were normalized to β-Actin.

### 4.6. MILLIPLEX^®^ MAP Human Glycolysis Pathway Magnetic Bead Panel Assays

The MILLIPLEX^®^ MAP Human Glycolysis Pathway Magnetic Bead Panel (HGPMAG-27K, Millipore, Burlington, MA, USA) was applied in 96-well plates for the simultaneous quantification of the following proteins in tissue lysates: G6PI (glucose-6-phosphate isomerase), LDHA (L-lactate dehydrogenase A chain), LDHB (L-lactate dehydrogenase B chain), PKM2 (pyruvate kinase isoform M2), TKT (transketolase) and HIF-1α (hypoxia-inducible factor 1-α).

For the immunoassay procedures, 25 µL of each dilute lysate sample in assay buffer (5 µg total protein/well) and HeLa cell lysate (positive control) were added into wells in duplicate, according to the manufacturer’s instructions. To each well, 25 µL of the Mixed Beads were added and the plate was incubated for 2 h at room temperature. Human glycolysis pathway detection biotinylated antibodies were added for 1 h; each captured a specific bead. After that, the reaction mixture was incubated for 30 min with Streptavidin-PE conjugate to complete the reaction on the surface of each microsphere. Finally, the MILLIPLEX^®^ MAP was analyzed by Luminex xMAP^®^ technology. The immunoassay on the surface of each fluorescent-coded magnetic bead, MagPlex-C microsphere, was identified and quantified based on fluorescent signals. The median fluorescence intensity (MFI) was read with the Luminex 200TM instrument and measured with xPONENT^®^ software.

### 4.7. Determination of Glucose-6-Phosphate Dehydrogenase Activity

Glucose-6-phosphate dehydrogenase (G6PDH) activity was assessed in tissue lysates using the Glucose-6-Phosphate Dehydrogenase Assay Kit (Abcam, Cambridge, UK). The assay was performed by adding 50 μL of dilute samples (1:2 with assay buffer) into duplicate wells of 96-well plates. Then, 50 μL of reaction mix (G6PDH substrate and G6PDH developer) was added to each well containing samples or positive control. At two (T1) and thirty (T2) minutes after addition of the reagents and incubation at 37 °C, the plate was read in an ELISA microplate reader at 450 nm. G6PDH activity was spectrophotometrically measured by monitoring the production of NADH, and calculated as a change in optical density (ΔA450 nm) at T1 and T2 per sample volume added into the reaction well, expressed in nmol/mg.

### 4.8. Primary Cell Cultures from Renal Tissues

Tumor (ccRCC) and normal (N) kidney tissue specimens were immediately placed in a Petri dish with phosphate-buffered saline (PBS) 1× and cut into small pieces of about 1 mm^3^. Each small piece of tissue was placed on the surface of the Petri dish with Dulbecco’s Modified Eagle Medium (DMEM, Invitrogen, Life Technologies, Monza, Italy) supplemented with 10% fetal bovine serum (FBS, Sigma-Aldrich, Milan, Italy) and 1% penicillin-streptomycin-L-glutamine (Sigma-Aldrich, Milan, Italy). Cell proliferation was obtained around the kidney specimens as previously described [55]. Kidney epithelial tubular and neoplastic cells were isolated with EpCAM (CD326) Ab-conjugated magnetic microbeads (Miltenyi Biotec, Bergisch Gladbach, Germany) under the effect of a magnetic field generated by the Mini MACS Separation Unit (Miltenyi Biotec), as previously described [55]. These cells were then characterized for EpCAM, CA IX and MUC1 by immunocytochemistry. Primary ccRCC cells were used for proliferation studies at the second passage.

### 4.9. Small Interfering (siRNA) Transfection

Isolated normal and tumor renal cells were cultured at 2 × 105 cells per well in a 12-well plate with Keratinocyte Serum-Free Medium (KSFM), supplemented with 5 ng/mL recombinant epidermal growth factor (rEGF), 50 µg/mL bovine pituitary extract (BPE) (Gibco, Life Technologies, Monza, Italy) and 30 ng/mL cholera toxin (Sigma-Aldrich, Milan, Italy). The transfection of siRNA was carried out using Lipofectamine 3000 (Life Technologies, Monza, Italy) in accordance with the manufacturer’s procedure. For each transfection, 50 nM of small interfering RNA targeting MUC1 (siMUC1) (Qiagen, Hilden, Germany) was used. In transfection experiments, a mock transfection control was performed by putting cells through the transfection procedure without adding siRNA. The validated non-silencing siRNA sequence AllStars Negative Control siRNA (50 nM, Qiagen, Hilden, Germany) was used as negative control. Each transfection experiment was performed in triplicate. After transfection, normal and tumor renal cells were incubated for 12 h and 24 h at 37 °C in 5% CO_2_ and used for total RNA extraction, wound-healing and cell viability assays.

### 4.10. Immunohistochemistry

Immunohistochemical evaluation of MUC1, GLUT1 and NDUFA4L2 protein expression was conducted on paraffin-embedded tissue sections. These sections (3 mm) were deparaffinized and rehydrated through xylenes and graded alcohol series. Slides were subjected to specific epitope demasking by microwave treatment at 700 W in citrate buffer (0.01 M, pH 6.0). After antigen retrieval, the tissue samples were incubated for 10 min with 3% H_2_O_2_ to block endogenous peroxidase activity. Sections were blocked with Protein Block Serum-Free (Dako) at room temperature for 10 min and then incubated with anti-MUC1 antibody (1:200, Novus Biologicals, Littleton, CO, USA), anti-NDUFA4L2 antibody (1:100 dilution, Proteintech, Chicago, IL, USA) and anti-GLUT1 antibody (1:200, Novus Biologicals, Littleton, CO, USA) at 4 °C overnight. Binding of the secondary biotinylated antibody was detected using the Dako Real EnVision Detection System, Peroxidase/DAB kit (Dako, Agilent, Santa Clara, CA, USA), according to the manufacturer’s instructions. Sections were counterstained with Mayer’s hematoxylin (blue) and mounted with glycerol (Dako Cytomation). Negative controls were obtained by incubating serial sections with the blocking solution and then omitting the primary antibodies. Digital images were obtained using the Aperio ScanScope CS2 device (Aperio Technologies, Vista, CA, USA) and further analyses of the scanned images were performed with the ImageScope V12.1.0.5029 (Aperio). Specific staining was quantified by applying the Positive Pixel Count v9_v10.0.0.1805 algorithm (Aperio) and expressed as percentage of positive pixels in the analyzed area.

### 4.11. Wound-Healing Assay

The 2 × 10^5^ normal and primary tumor renal cells were seeded onto a six-well plate with 2 mL of Keratinocyte Serum-Free Medium (KSFM) to create a confluent monolayer, supplemented with 5 ng/mL recombinant epidermal growth factor (rEGF), 50 μg/mL bovine pituitary extract (BPE) (Gibco) and 30 ng/mL cholera toxin (Sigma-Aldrich, Milan, Italy). Cells were incubated overnight at 37 °C, 5% CO_2_, and then exposed to 50 nM of siMUC1 (Quiagen) or incubated in medium without siRNA for 72 h. After 24 h from the treatment, a wound was manually created by scraping the cell monolayer with a P200 pipette tip. A reference mark was created on the dish and a time 0 image was acquired. After 12 and 24 h, additional images were taken in the matched region, and the wound-healing area was quantified with ImageJ software (http://rsbweb.nih.gov/ij/ (accessed on 1 March 2022)). Each experimental condition was performed in triplicate.

### 4.12. Cell Proliferation and Viability Assay 

Cell proliferation and viability assay, after exposure to 50 nM of siMUC1 or to siMUC1 and 10 μM cis-Diamminedichloroplatinum(II) (cisplatin), was evaluated using the trypan blue dye exclusion and 3-(4,5-dimethylthiazol-2-yl)-2,5-diphenyltetrazolium bromide (MTT) assay.

For the dye exclusion test and MTT assay, normal and primary tumor cells were seeded at a density of 1.5 × 105 and 1.5 × 104 cells in 6-well and 96-well plates, respectively, (Sigma Aldrich, Milan, Italy) and incubated overnight at 37 °C, 5% CO_2_, 1 day before exposure to siMUC1 or to medium alone. The cells were exposed to 50 nM of siMUC1 for 24 h and then treated with 10 μM cis-Diamminedichloroplatinum(II) (cisplatin) for 1 h and 2 h. After several washes to remove cisplatin, the cells were again incubated in medium with 50 nM of siMUC1 for 48 h. Lastly, the cells were trypsinized and the viable cells were counted using trypan blue or were exposed to MTT. Each experimental condition was performed in triplicate.

### 4.13. Immunofluorescence Microscopy

Primary tumor cells were seeded at a density of 2 × 10^5^ on glass coverslips and left to adhere overnight at 37 °C in 5% CO_2_. These preparations were stained for NDUFA4L2 (Proteintech, Chicago, IL, USA) and MitoSOX red (Molecular Probes, Life Technologies, Monza, Italy). The expression and localization of proteins was evaluated by indirect immunofluorescence and confocal microscopy analysis. In particular, the cells were fixed using ice-cold 4% paraformaldehyde for 10 min at room temperature. Then, the preparations were blocked with 1% BSA in PBS for 1 h at room temperature and incubated overnight at 4 °C with a primary antibody against NDUFA4L2 (1:25 in blocking), followed by incubation for 1 h at 37 °C with the secondary antibody goat anti-rabbit IgG FITC (1:200; Novus Biologicals). To study mitochondrial superoxide generation, live cells were stained with 5 μM MitoSOX red for 10 min at 37 °C. All preparations were counterstained with TO-PRO-3 (Molecular Probes). Negative controls were performed by omitting the primary antibodies. Specific fluorescence was acquired by a Leica TCS SP2 (Leica, Wetzlar, Germany) confocal laser-scanning microscope using a ×63 objective lens.

### 4.14. Mitochondrial Membrane Potential

Mitochondrial membrane potential was determined by incubating primary tumor cells with the fluorescent dye tetramethylrhodamine ethyl ester (TMRE; Sigma-Aldrich, Milan, Italy). Cells were incubated with 2.5 µM TMRE for 1 h at 37 °C and subsequently analyzed by immunofluorescence.

## Figures and Tables

**Table 1:** Univariate and multivariate analyses for cancer-specific survival. CI: confidence interval; HR: hazard ratio.

Variable	Category	Univariate Analysis				Multivariate Analysis			
		CI 95%				CI 95%			
		HR	Lower	Higher	*p*-Value	HR	Lower	Higher	*p*-Value
T stage	T3–4 vs. T1–2	2.11	1.54	2.66	0.0001	1.36	1.12	2.09	0.001
N stage	N+ vs. N0	2.84	1.94	5.36	0.001	1.28	1.11	2.59	0.01
M stage	M+ vs. M0	5.21	2.93	9.44	0.0001	3.95	2.21	8.42	0.001
Grade	G3–4 vs. G1–2	2.14	1.12	5.76	0.01	1.61	1.14	2.25	0.01
Necrosis	Yes vs. No	2.34	1.21	3.87	0.01	-	-	-	-
Tumor size	Continuous	1.47	1.09	2.13	0.01	-	-	-	-
CA15-3	>25 vs. ≤25 U/mL	2.21	1.28	5.31	0.001	1.97	1.08	2.94	0.01

**Table 2:** Univariate and multivariate analyses for progression-free survival. CI: confidence interval; HR: hazard ratio.

Variable	Category	Univariate Analysis				Multivariate Analysis			
		CI 95%				CI 95%			
		HR	Lower	Higher	*p*-Value	HR	Lower	Higher	*p*-Value
T stage	T3–4 vs. T1–2	2.24	1.33	2.75	0.001	1.98	1.24	2.95	0.01
N stage	N+ vs. N0	2.96	1.94	6.25	0.001	2.18	1.16	2.96	0.01
M stage	M+ vs. M0	6.01	2.43	10.25	0.001	3.15	2.13	7.12	0.001
Grade	G3–4 vs. G1–2	2.11	1.94	6.54	0.01	1.71	1.24	3.21	0.01
Necrosis	Yes vs. No	2.37	1.19	3.04	0.01	-	-	-	-
Tumor size	Continuous	1.86	1.07	2.69	0.01	-	-	-	-
CA15-3	>25 vs. ≤25 U/mL	2.35	1.18	4.76	0.001	1.95	1.04	2.85	0.01

## Data Availability

The datasets generated and/or analyzed during the current study are available in the GEO repository: accession number GSE15641.

## Supplementary Materials

The following supporting information can be downloaded at: https://www.mdpi.com/article/10.3390/ijms232213968/s1.

## References

[1] Siegel R.L., Miller K.D., Fuchs H.E., Jemal A. (2022). Cancer statistics, 2022. CA Cancer J. Clin..

[2] Lucarelli G., Loizzo D., Franzin R., Battaglia S., Ferro M., Cantiello F., Castellano G., Bettocchi C., Ditonno P., Battaglia M. (2019). Metabolomic insights into pathophysiological mechanisms and biomarker discovery in clear cell renal cell carcinoma. Expert Rev. Mol. Diagn..

[3] Lucarelli G., Ferro M., Battaglia M. (2016). Multi-omics approach reveals the secrets of metabolism of clear cell-renal cell carcinoma. Transl. Androl. Urol..

[4] Lucarelli G., Ferro M., Ditonno P., Battaglia M. (2018). The urea cycle enzymes act as metabolic suppressors in clear cell renal cell carcinoma. Transl. Cancer Res..

[5] Ragone R., Sallustio F., Piccinonna S., Rutigliano M., Vanessa G., Palazzo S., Lucarelli G., Ditonno P., Battaglia M., Fanizzi F.P. (2016). Renal Cell Carcinoma: A Study through NMR-Based Metabolomics Combined with Transcriptomics. Diseases.

[6] Lucarelli G., Fanelli M., Larocca A.M., Germinario C.A., Rutigliano M., Vavallo A., Selvaggi F.P., Bettocchi C., Battaglia M., Ditonno P. (2012). Serum sarcosine increases the accuracy of prostate cancer detection in patients with total serum PSA less than 4.0 ng/mL. Prostate.

[7] Lucarelli G., Ditonno P., Bettocchi C., Spilotros M., Rutigliano M., Vavallo A., Galleggiante V., Fanelli M., Larocca A.M., Germinario C.A. (2013). Serum sarcosine is a risk factor for progression and survival in patients with metastatic castration-resistant prostate cancer. Future Oncol..

[8] di Meo N.A., Loizzo D., Pandolfo S.D., Autorino R., Ferro M., Porta C., Stella A., Bizzoca C., Vincenti L., Crocetto F. (2022). Metabolomic Approaches for Detection and Identification of Biomarkers and Altered Pathways in Bladder Cancer. Int. J. Mol. Sci..

[9] Lucarelli G., Loizzo D., Ferro M., Rutigliano M., Vartolomei M.D., Cantiello F., Buonerba C., Di Lorenzo G., Terracciano D., De Cobelli O. (2019). Metabolomic profiling for the identification of novel diagnostic markers and therapeutic targets in prostate cancer: An update. Expert Rev. Mol. Diagn..

[10] Cancer Genome Atlas Research Network (2013). Comprehensive molecular characterization of clear cell renal cell carcinoma. Nature.

[11] Ricketts C.J., De Cubas A.A., Fan H., Smith C.C., Lang M., Reznik E., Bowlby R., Gibb E.A., Akbani R., Beroukhim R. (2018). The Cancer Genome Atlas Comprehensive Molecular Characterization of Renal Cell Carcinoma. Cell Rep..

[12] Hakimi A.A., Reznik E., Lee C.H., Creighton C.J., Brannon A.R., Luna A., Aksoy B.A., Liu E.M., Shen R., Lee W. (2016). An Integrated Metabolic Atlas of Clear Cell Renal Cell Carcinoma. Cancer Cell.

[13] Petroniene J., Morkvenaite-Vilkonciene I., Miksiunas R., Bironaite D., Ramanaviciene A., Rucinskas K., Janusauskas V., Ramanavicius A. (2020). Scanning electrochemical microscopy for the investigation of redox potential of human myocardium-derived mesenchymal stem cells grown at 2D and 3D conditions. Electrochim. Acta.

[14] Lucarelli G., Galleggiante V., Rutigliano M., Sanguedolce F., Cagiano S., Bufo P., Lastilla G., Maiorano E., Ribatti D., Giglio A. (2015). Metabolomic profile of glycolysis and the pentose phosphate pathway identifies the central role of glucose-6-phosphate dehydrogenase in clear cell-renal cell carcinoma. Oncotarget.

[15] Bombelli S., Torsello B., De Marco S., Lucarelli G., Cifola I., Grasselli C., Strada G., Bovo G., Perego R.A., Bianchi C. (2020). 36-kDa Annexin A3 Isoform Negatively Modulates Lipid Storage in Clear Cell Renal Cell Carcinoma Cells. Am. J. Pathol..

[16] Lucarelli G., Ferro M., Loizzo D., Bianchi C., Terracciano D., Cantiello F., Bell L.N., Battaglia S., Porta C., Gernone A. (2020). Integration of Lipidomics and Transcriptomics Reveals Reprogramming of the Lipid Metabolism and Composition in Clear Cell Renal Cell Carcinoma. Metabolites.

[17] Lucarelli G., Rutigliano M., Sallustio F., Ribatti D., Giglio A., Lepore Signorile M., Grossi V., Sanese P., Napoli A., Maiorano E. (2018). Integrated multi-omics characterization reveals a distinctive metabolic signature and the role of NDUFA4L2 in promoting angiogenesis, chemoresistance, and mitochondrial dysfunction in clear cell renal cell carcinoma. Aging.

[18] Battaglia M., Lucarelli G. (2015). The role of renal surgery in the era of targeted therapy: The urologist’s perspective. Urologia.

[19] Monti M., Lunardini S., Magli I.A., Campi R., Primiceri G., Berardinelli F., Amparore D., Terracciano D., Lucarelli G., Schips L. (2022). Micro-RNAs Predict Response to Systemic Treatments in Metastatic Renal Cell Carcinoma Patients: Results from a Systematic Review of the Literature. Biomedicines.

[20] Lucarelli G., Ditonno P., Bettocchi C., Vavallo A., Rutigliano M., Galleggiante V., Larocca A.M., Castellano G., Gesualdo L., Grandaliano G. (2014). Diagnostic and prognostic role of preoperative circulating CA15-3, CA 125, and beta-2 microglobulin in renal cell carcinoma. Dis. Markers.

[21] Lucarelli G., Rutigliano M., Sanguedolce F., Galleggiante V., Giglio A., Cagiano S., Bufo P., Maiorano E., Ribatti D., Ranieri E. (2015). Increased Expression of the Autocrine Motility Factor is Associated with Poor Prognosis in Patients with Clear Cell-Renal Cell Carcinoma. Medicine.

[22] Gigante M., Lucarelli G., Divella C., Netti G.S., Pontrelli P., Cafiero C., Grandaliano G., Castellano G., Rutigliano M., Stallone G. (2015). Soluble Serum αKlotho is a Potential Predictive Marker of Disease Progression in Clear Cell Renal Cell Carcinoma. Medicine.

[23] Papale M., Vocino G., Lucarelli G., Rutigliano M., Gigante M., Rocchetti M.T., Pesce F., Sanguedolce F., Bufo P., Battaglia M. (2017). Urinary RKIP/p-RKIP is a potential diagnostic and prognostic marker of clear cell renal cell carcinoma. Oncotarget.

[24] Netti G.S., Lucarelli G., Spadaccino F., Castellano G., Gigante M., Divella C., Rocchetti M.T., Rascio F., Mancini V., Stallone G. (2020). PTX3 modulates the immunoflogosis in tumor microenvironment and is a prognostic factor for patients with clear cell renal cell carcinoma. Aging.

[25] Bialek J., Wencker A., Kawan F., Yankulov S., Fornara P., Theil G. (2022). Potential Use of CTCs as Biomarkers in Renal Cancer Patients. Life.

[26] Chen W., Zhang Z., Zhang S., Zhu P., Ko J., Yung K. (2021). MUC1: Structure, Function, and Clinic Application in Epithelial Cancers. Int. J. Mol. Sci..

[27] Horm T.M., Schroeder J.A. (2013). MUC1 and metastatic cancer: Expression, function and therapeutic targeting. Cell Adh. Migr..

[28] Beckwith D.M., Cudic M. (2020). Tumor-associated O-glycans of MUC1: Carriers of the glyco-code and targets for cancer vaccine design. Semin. Immunol..

[29] Gao T., Cen Q., Lei H. (2020). A review on development of MUC1-based cancer vaccine. Biomed. Pharmacother..

[30] Mehla K., Singh P.K. (2014). MUC1: A novel metabolic master regulator. Biochim. Biophys. Acta.

[31] Kraus S., Abel P.D., Nachtmann C., Linsenmann H.-J., Weidner W., Stamp G.W.H., Chaudhary K.S., Mitchell S.E., Franke F.E., Lalani E.-N. (2002). MUC1 mucin and trefoil factor 1 protein expression in renal cell carcinoma: Correlation with prognosis. Hum. Pathol..

[32] Leroy X., Zerimech F., Zini L., Copin M.-C., Buisine M.-P., Gosselin B., Aubert J.-P., Porchet N. (2002). MUC1 expression is correlated with nuclear grade and tumor progression in pT1 renal clear cell carcinoma. Am. J. Clin. Pathol..

[33] Langner C., Ratschek M., Rehak P., Schips L., Zigeuner R. (2004). Expression of MUC1 (EMA) and E-cadherin in renal cell carcinoma: A systematic immunohistochemical analysis of 188 cases. Mod. Pathol..

[34] Aubert S., Fauquette V., Hémon B., Lepoivre R., Briez N., Bernard D., Van Seuningen I., Leroy X., Perrais M. (2009). MUC1, a new hypoxia inducible factor target gene, is an actor in clear renal cell carcinoma tumor progression. Cancer Res..

[35] Chong J., Xia J. (2020). Using MetaboAnalyst 4.0 for Metabolomics Data Analysis, Interpretation, and Integration with Other Omics Data. Methods Mol. Biol..

[36] Chaika N.V., Gebregiworgis T., Lewallen M.E., Purohit V., Radhakrishnan P., Liu X., Zhang B., Mehla K., Brown R.B., Caffrey T. (2012). MUC1 mucin stabilizes and activates hypoxia-inducible factor 1 alpha to regulate metabolism in pancreatic cancer. Proc. Natl. Acad. Sci. USA.

[37] Mullen A.R., Wheaton W.W., Jin E.S., Chen P., Sullivan L.B., Cheng T., Yang Y., Linehan W.M., Chandel N.S., DeBerardinis R.J. (2011). Reductive carboxylation supports growth in tumour cells with defective mitochondria. Nature.

[38] Lucarelli G., Rutigliano M., Ferro M., Giglio A., Intini A., Triggiano F., Palazzo S., Gigante M., Castellano G., Ranieri E. (2017). Activation of the kynurenine pathway predicts poor outcome in patients with clear cell renal cell carcinoma. Urol. Oncol..

[39] Bianchi C., Meregalli C., Bombelli S., Di Stefano V., Salerno F., Torsello B., De Marco S., Bovo G., Cifola I., Mangano E. (2017). The glucose and lipid metabolism reprogramming is grade-dependent in clear cell renal cell carcinoma primary cultures and is targetable to modulate cell viability and proliferation. Oncotarget.

[40] Subramanian A., Tamayo P., Mootha V.K., Mukherjee S., Ebert B.L., Gillette M.A., Paulovich A., Pomeroy S.L., Golub T.R., Lander E.S. (2005). Gene set enrichment analysis: A knowledge-based approach for interpreting genome-wide expression profiles. Proc. Natl. Acad. Sci. USA.

[41] Reynolds I.S., Fichtner M., McNamara D.A., Kay E.W., Prehn J.H., Burke J.P. (2019). Mucin glycoproteins block apoptosis; promote invasion, proliferation, and migration; and cause chemoresistance through diverse pathways in epithelial cancers. Cancer Metastasis Rev..

[42] Nath S., Daneshvar K., Roy L.D., Grover P., Kidiyoor A., Mosley L., Sahraei M., Mukherjee P. (2013). MUC1 induces drug resistance in pancreatic cancer cells via upregulation of multidrug resistance genes. Oncogenesis.

[43] Shukla S.K., Purohit V., Mehla K., Gunda V., Chaika N.V., Vernucci E., King R.J., Abrego J., Goode G.D., Dasgupta A. (2017). MUC1 and HIF-1alpha Signaling Crosstalk Induces Anabolic Glucose Metabolism to Impart Gemcitabine Resistance to Pancreatic Cancer. Cancer Cell.

[44] Duffy M.J., Evoy D., McDermott E.W. (2010). CA15-3: Uses and limitation as a biomarker for breast cancer. Clin. Chim. Acta.

[45] Gunda V., Souchek J., Abrego J., Shukla S.K., Goode G.D., Vernucci E., Dasgupta A., Chaika N.V., King R.J., Li S. (2017). MUC1-Mediated Metabolic Alterations Regulate Response to Radiotherapy in Pancreatic Cancer. Clin. Cancer Res..

[46] Yamamoto M., Jin C., Hata T., Yasumizu Y., Zhang Y., Hong D., Maeda T., Miyo M., Hiraki M., Suzuki Y. (2019). MUC1-C Integrates Chromatin Remodeling and PARP1 Activity in the DNA Damage Response of Triple-Negative Breast Cancer Cells. Cancer Res..

[47] Goode G., Gunda V., Chaika N.V., Purohit V., Yu F., Singh P.K. (2017). MUC1 facilitates metabolomic reprogramming in triple-negative breast cancer. PLoS ONE.

[48] Bian X., Liu R., Meng Y., Xing D., Xu D., Lu Z. (2021). Lipid metabolism and cancer. J. Exp. Med..

[49] De Marco S., Torsello B., Minutiello E., Morabito I., Grasselli C., Bombelli S., Zucchini N., Lucarelli G., Strada G., Perego R.A. (2022). The cross-talk between Abl2 tyrosine kinase and TGFβ1 signalling modulates the invasion of clear cell renal cell carcinoma cells. FEBS Lett..

[50] Wu Q., Yang Z., Nie Y., Shi Y., Fan D. (2014). Multi-drug resistance in cancer chemotherapeutics: Mechanisms and lab approaches. Cancer Lett..

[51] Choi Y.H., Yu A.M. (2014). ABC transporters in multidrug resistance and pharmacokinetics, and strategies for drug development. Curr. Pharm. Des..

[52] Tobe S.W., Noble-Topham S.E., Andrulis I.L., Hartwick R.W., Skorecki K.L., Warner E. (1995). Expression of the multiple drug resistance gene in human renal cell carcinoma depends on tumor histology, grade, and stage. Clin. Cancer Res..

[53] Grankvist K., Ljungberg B., Rasmuson T. (1997). Evaluation of five glycoprotein tumour markers (CEA, CA-50, CA-19-9, CA-125, CA-15-3) for the prognosis of renal-cell carcinoma. Int. J. Cancer.

[54] Briasoulis E., Pentheroudakis G., Letsa I., Pavlidis N. (2002). A retrospective analysis of serum CA15-3 concentrations in patients with localised or metastatic renal cancer and its impact on prognosis and follow-up. A single-centre experience. UroOncology.

[55] Galleggiante V., Rutigliano M., Sallustio F., Ribatti D., Ditonno P., Bettocchi C., Selvaggi F.P., Lucarelli G., Battaglia M. (2014). CTR2 identifies a population of cancer cells with stem cell-like features in patients with clear cell renal cell carcinoma. J. Urol..

