# Supplementary material for: MUC1 Tissue Expression and Its Soluble Form CA15-3 Identify a Clear Cell Renal Cell Carcinoma with Distinct Metabolic Profile and Poor Clinical Outcome
Source: Int J Mol Sci. 2022 Nov 12;23(22):13968. doi: 10.3390/ijms232213968 (PMC9696833; doi:10.3390/ijms232213968)
Supplement: Supplementary file 1 [file ijms-23-13968-s001.zip › Supplementary Figure S3.pdf]

Pathway integrative analysis

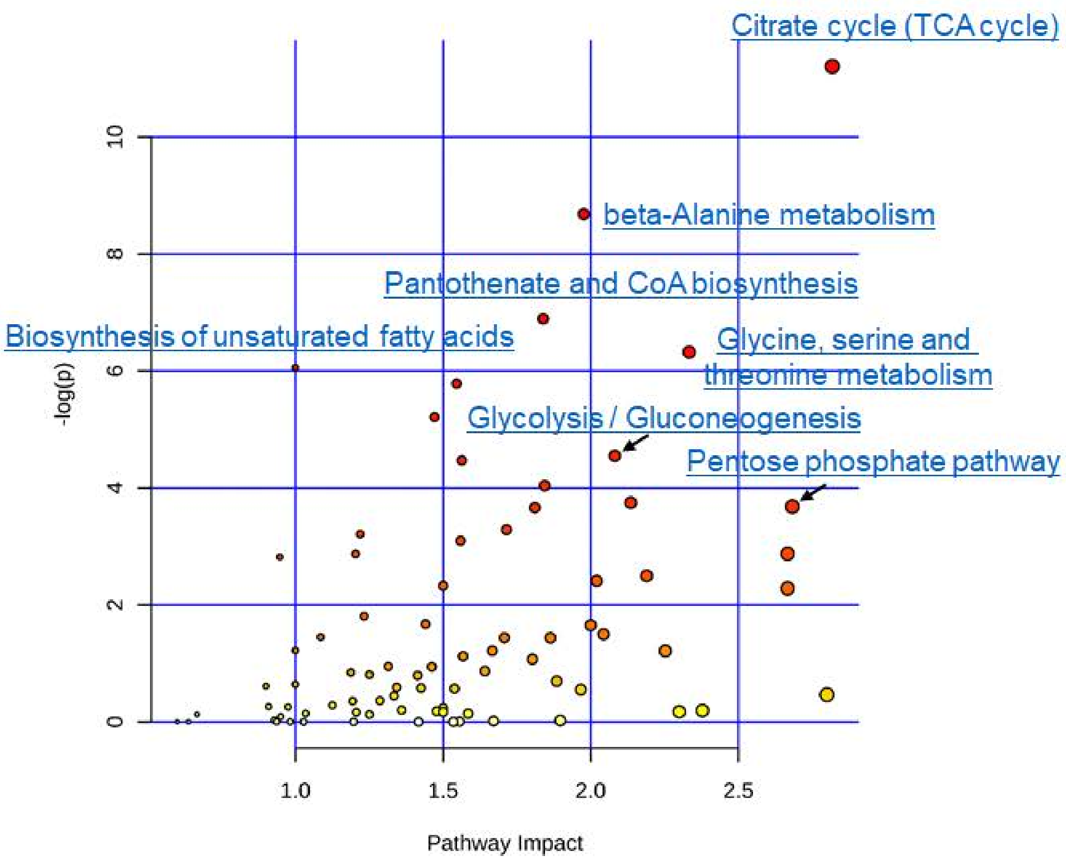

Pathway Name	P-value	FDR
Citrate cycle (TCA cycle)	1.3586E-5	0.0010869
beta-Alanine metabolism	1.6945E-4	0.006778
Pantothenate and CoA biosynthesis	0.0010159	0.02709
Glycine, serine and threonine metabolism	0.0017954	0.035908
Biosynthesis of unsaturated fatty acids	0.0023464	0.037542
Valine, leucine and isoleucine biosynthesis	0.0030806	0.041075
Caffeine metabolism	0.0054486	0.06227
Alanine, aspartate and glutamate metabolism	0.010563	0.10164
Cysteine and methionine metabolism	0.011435	0.10164
Butanoate metabolism	0.017607	0.14086
Glycolysis / Gluconeogenesis	0.023546	0.15784
Pentose phosphate pathway	0.025192	0.15784
Fructose and mannose metabolism	0.025649	0.15784
D-Glutamine and D-glutamate metabolism	0.037392	0.21367
Inositol phosphate metabolism	0.040297	0.21492
Arginine and proline metabolism	0.045196	0.22598
