# Supplementary material for: MUC1 Tissue Expression and Its Soluble Form CA15-3 Identify a Clear Cell Renal Cell Carcinoma with Distinct Metabolic Profile and Poor Clinical Outcome
Source: Int J Mol Sci. 2022 Nov 12;23(22):13968. doi: 10.3390/ijms232213968 (PMC9696833; doi:10.3390/ijms232213968)
Supplement: Supplementary file 1 [file ijms-23-13968-s001.zip › Supplementary Table S1.pdf]

**Variable**	**n=36**
Age (years)<br>median<br>range	 61<br>27-82
Gender<br>Male<br>Female	 23 (63.8%)<br>13 (36.2%)
Dimensions (cm)<br>median<br>range	 5.0<br>3 -10
Pathological stage<br>pT1<br>pT2<br>pT3	 22 (62%)<br>9 (25%)<br>5 (13%)
pN+	7 (19.4%)
cM+	3 (8.3%)
Fuhrman grade<br>G1-2<br>G3-4	 24 (66.7%)<br>12 (33.3%)

Table S1: Clinical and pathological characteristics of tissues collected from patients who underwent radical or partial nephrectomy for ccRCC. These specimens were used for metabolomics analysis and to obtain primary cell cultures.
