# Supplementary material for: MUC1 Tissue Expression and Its Soluble Form CA15-3 Identify a Clear Cell Renal Cell Carcinoma with Distinct Metabolic Profile and Poor Clinical Outcome
Source: Int J Mol Sci. 2022 Nov 12;23(22):13968. doi: 10.3390/ijms232213968 (PMC9696833; doi:10.3390/ijms232213968)
Supplement: Supplementary file 1 [file ijms-23-13968-s001.zip › Supplementary Table S2.pdf]

**Essential fatty acids**
dihomo-linolenate (20:3n3 or n6)<br>docosahexaenoate (DHA; 22:6n3)<br>docosapentaenoate (n3 DPA; 22:5n3)<br>docosapentaenoate (n6 DPA; 22:5n6)<br>eicosapentaenoate (EPA; 20:5n3)<br>linoleate (18:2n6)<br>linolenate [alpha or gamma; (18:3n3 or 6)]
**Medium chain fatty acids**
5-dodecenoate (12:1n7)<br>caprate (10:0)<br>laurate (12:0)<br>pelargonate (9:0)
**Long chain fatty acids**
10-heptadecenoate (17:1n7)<br>10-nonadecenoate (19:1n9)<br>adrenate (22:4n6)<br>arachidate (20:0)<br>arachidonate (20:4n6)<br>behenate (22:0)<br>cis-vaccenate (18:1n7)<br>dihomo-linoleate (20:2n6)<br>docosadienoate (22:2n6)<br>docosatrienoate (22:3n3)<br>eicosenoate (20:1n9 or 11)<br>erucate (22:1n9)<br>margarate (17:0)<br>myristate (14:0)<br>myristoleate (14:1n5)<br>nervonate (24:1n9)<br>nonadecanoate (19:0)<br>oleate (18:1n9)<br>palmitate (16:0)<br>palmitoleate (16:1n7)<br>pentadecanoate (15:0)<br>stearate (18:0)<br>stearidonate (18:4n3)
**Lysolipids**
1-arachidonoylglycerophosphocholine<br>1-arachidonoylglycerophosphoethanolamine<br>1-arachidonoylglycerophosphoinositol<br>1-eicosadienoylglycerophosphocholine<br>1-heptadecanoylglycerophosphocholine<br>1-heptadecanoylglycerophosphoethanolamine

1-linoleoylglycerophosphocholine<br>1-linoleoylglycerophosphoethanolamine<br>1-myristoylglycerophosphocholine<br>1-oleoylglycerophosphocholine<br>1-oleoylglycerophosphoethanolamine<br>1-oleoylglycerophosphoinositol<br>1-palmitoleoylglycerophosphocholine<br>1-palmitoylglycerophosphocholine<br>1-palmitoylglycerophosphoethanolamine<br>1-palmitoylglycerophosphoinositol<br>1-palmitoylplasmenylethanolamine<br>1-stearoylglycerophosphocholine<br>1-stearoylglycerophosphoethanolamine<br>1-stearoylglycerophosphoinositol<br>2-arachidonoylglycerophosphocholine<br>2-arachidonoylglycerophosphoethanolamine<br>2-arachidonoylglycerophosphoinositol<br>2-docosahexaenoylglycerophosphocholine<br>2-docosahexaenoylglycerophosphoethanolamine<br>2-docosapentaenoylglycerophosphoethanolamine<br>2-eicosapentaenoylglycerophosphoethanolamine<br>2-eicosatrienoylglycerophosphocholine<br>2-linoleoylglycerophosphocholine<br>2-linoleoylglycerophosphoethanolamine<br>2-myristoylglycerophosphocholine<br>2-oleoylglycerophosphocholine<br>2-oleoylglycerophosphoethanolamine<br>2-oleoylglycerophosphoinositol<br>2-oleoylglycerophosphoserine<br>2-palmitoleoylglycerophosphocholine<br>2-palmitoylglycerophosphocholine<br>2-palmitoylglycerophosphoethanolamine<br>2-stearoylglycerophosphocholine
**Neutral lipids**
11-dehydrocorticosterone<br>4-androsten-3beta,17beta-diol disulfate 1<br>7-alpha-hydroxy-3-oxo-4-cholestenoate (7-Hoca)<br>7-alpha-hydroxycholesterol<br>7-beta-hydroxycholesterol<br>andro steroid monosulfate 2<br>androsterone sulfate<br>cholesterol<br>cortisone<br>dehydroisoandrosterone sulfate (DHEA-S)<br>dihydrocholesterol<br>epiandrosterone sulfate

pregn steroid monosulfate<br>pregnanediol-3-glucuronide<br>pregnen-diol disulfate<br>squalene
**Sphingolipids**
galactosylsphingosine<br>palmitoyl sphingomyelin<br>phytosphingosine<br>sphinganine<br>sphingosine<br>stearoyl sphingomyelin
**Glycerolipids**
choline<br>choline phosphate<br>cytidine 5'-diphosphocholine<br>ethanolamine<br>glycerol<br>glycerol 3-phosphate (G3P)<br>glycerophosphoethanolamine<br>glycerophosphorylcholine (GPC)<br>phosphoethanolamine
**Carnitine metabolism**
3-dehydrocarnitine<br>acetylcarnitine<br>carnitine<br>decanoylcarnitine<br>deoxycarnitine<br>hexanoylcarnitine<br>laurylcarnitine<br>myristoylcarnitine<br>octanoylcarnitine<br>oleoylcarnitine<br>palmitoylcarnitine<br>stearoylcarnitine
**Eicosanoids**
13,14-dihydro-15-keto-prostaglandin a2<br>5-HETE<br>5-oxoETE<br>6-keto prostaglandin F1alpha<br>prostaglandin A2<br>prostaglandin E2<br>prostaglandin I2

Table S2: Detailed list of metabolites identified in each lipid category
