# Supplementary material for: MUC1 Tissue Expression and Its Soluble Form CA15-3 Identify a Clear Cell Renal Cell Carcinoma with Distinct Metabolic Profile and Poor Clinical Outcome
Source: Int J Mol Sci. 2022 Nov 12;23(22):13968. doi: 10.3390/ijms232213968 (PMC9696833; doi:10.3390/ijms232213968)
Supplement: Supplementary file 1 [file ijms-23-13968-s001.zip › Supplementary Table S3.pdf]

**Variable**	**n=428**
**Age (years)**<br>**median**<br>**95% CI**	 62<br>60-64
**Gender**<br>**Male**<br>**Female**	 270 (63%)<br>158 (37%)
**Dimensions (cm)**<br>**median**<br>**95% CI**	 4.5<br>4.0 - 5.0
**Pathological stage**<br>**pT1a**<br>**pT1b**<br>**pT2**<br>**pT3**<br>**pT4**	 164 (38%)<br>110 (26%)<br>77 (18%)<br>54 (13%)<br>23 (5%)
**pN+**	32 (7.5%)
**cM+**	34 (7.9%)
**Fuhrman grade**<br>**G1-2**<br>**G3-4**	 306 (63%)<br>122 (37%)
**CA15-3 (U/mL)**<br>**Median**<br>**95% CI**<br>**range**	 23.8<br>21.8 – 27.1<br>9.7 – 115.7

Table S3: Clinical and pathological characteristics of patients enrolled for CA15-3 levels evaluation
