# Supplementary material for: MUC1 Tissue Expression and Its Soluble Form CA15-3 Identify a Clear Cell Renal Cell Carcinoma with Distinct Metabolic Profile and Poor Clinical Outcome
Source: Int J Mol Sci. 2022 Nov 12;23(22):13968. doi: 10.3390/ijms232213968 (PMC9696833; doi:10.3390/ijms232213968)
Supplement: Supplementary file 1 [file ijms-23-13968-s001.zip › Supplementary Table S4.pdf]

Type	Description	Purpose
MTRX	Large pool of human plasma maintained by Metabolon that has been characterized extensively.	Assure that all aspects of Metabolon process are operating within specifications.
CMTRX	Pool created by taking a small aliquot from every customer sample.	Assess the effect of a non-plasma matrix on the Metabolon process and distinguish biological variability from process variability.
PRCS	Aliquot of ultra-pure water	Process Blank used to assess the contribution to compound signals from the process.
SOLV	Aliquot of solvents used in extraction.	Solvent blank used to segregate contamination sources in the extraction.

Table S4. Description of Metabolon QC Samples.
