# Supplementary material for: MUC1 Tissue Expression and Its Soluble Form CA15-3 Identify a Clear Cell Renal Cell Carcinoma with Distinct Metabolic Profile and Poor Clinical Outcome
Source: Int J Mol Sci. 2022 Nov 12;23(22):13968. doi: 10.3390/ijms232213968 (PMC9696833; doi:10.3390/ijms232213968)
Supplement: Supplementary file 1 [file ijms-23-13968-s001.zip › Supplementary Table S5.pdf]

Type	Description	Purpose
DS	Derivatization Standard	Assess variability of derivatization for GC/MS samples.
IS	Internal Standard	Assess variability and performance of instrument.
RS	Recovery Standard	Assess variability and verify performance of extraction and instrumentation.

Table S5. Metabolon QC Standards.
