# Supplementary material for: MUC1 Tissue Expression and Its Soluble Form CA15-3 Identify a Clear Cell Renal Cell Carcinoma with Distinct Metabolic Profile and Poor Clinical Outcome
Source: Int J Mol Sci. 2022 Nov 12;23(22):13968. doi: 10.3390/ijms232213968 (PMC9696833; doi:10.3390/ijms232213968)
Supplement: Supplementary file 1 [file ijms-23-13968-s001.zip › Supplementary Table S6.pdf]

G6PDH	5’-GAGGCTGCAGTTCCATGATG-3’<br>5-’GACTCCTCGGGGTTGAAGAA-3’
G6PI	5’-GATCCTCCTGGCCAACTTCT-3’<br>5’-GTTGGTTGGGCGATTTCCTT-3’
LDHA	5’-CAACATGGCAGCCTTTTCCT-3’<br>5’-CACGTTACGCTGGACCAAAT-3’
LDHB	5’-TCTGGGAAAGTCTCTGGCTG-3’<br>5’-ACTCTCCCCTTCTTGCTGAC-3’
PKM2	5’-CAGCAAGAAGGGTGTGAACC-3’<br>5’-ATGCCTTGCGGATGAATGAC-3’
TKT	5’-GCCTTTGACCAGATTCGCAT-3’<br>5’-CTCTGTAGCAACGCCATCAC-3’
HIF-1α	5’-TCCAAGAAGCCCTAACGTGT-3’<br>5’-TGATCGTCTGGCTGCTGTAA-3’
B-ACTIN	5’-AATCTGGCACCACACCTTCT-3’<br>5’-AGCCTGGATAGCAACGTACA-3’

Table S6: Primers used for real time PCR
