# Supplementary figures and images for: MUC1 Tissue Expression and Its Soluble Form CA15-3 Identify a Clear Cell Renal Cell Carcinoma with Distinct Metabolic Profile and Poor Clinical Outcome
Source: Int J Mol Sci. 2022 Nov 12;23(22):13968. doi: 10.3390/ijms232213968 (PMC9696833; doi:10.3390/ijms232213968)

GLUT<sub>1</sub>

MUC<sub>1</sub><sup>H</sup>

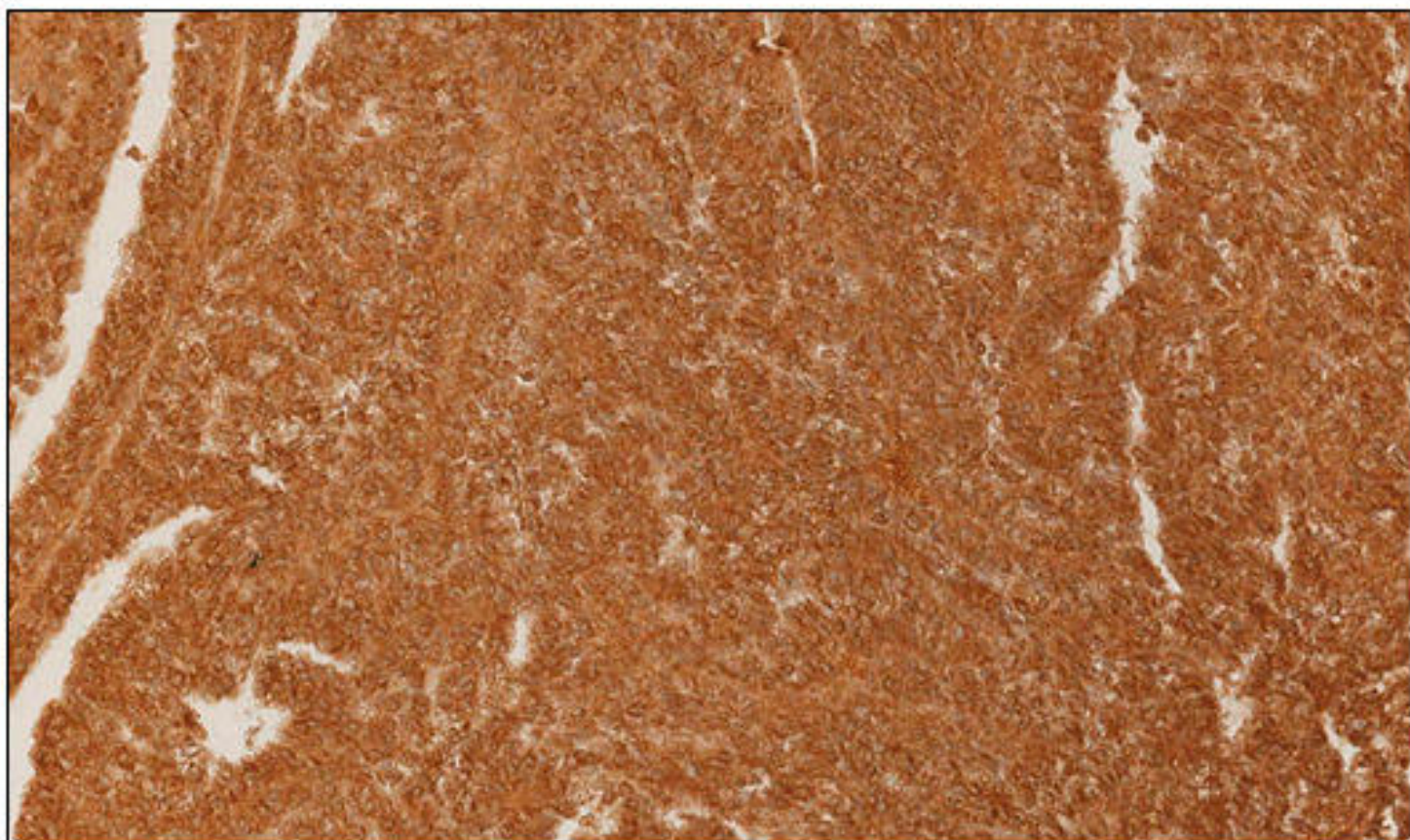

MUC<sub>1</sub><sup>L</sup>

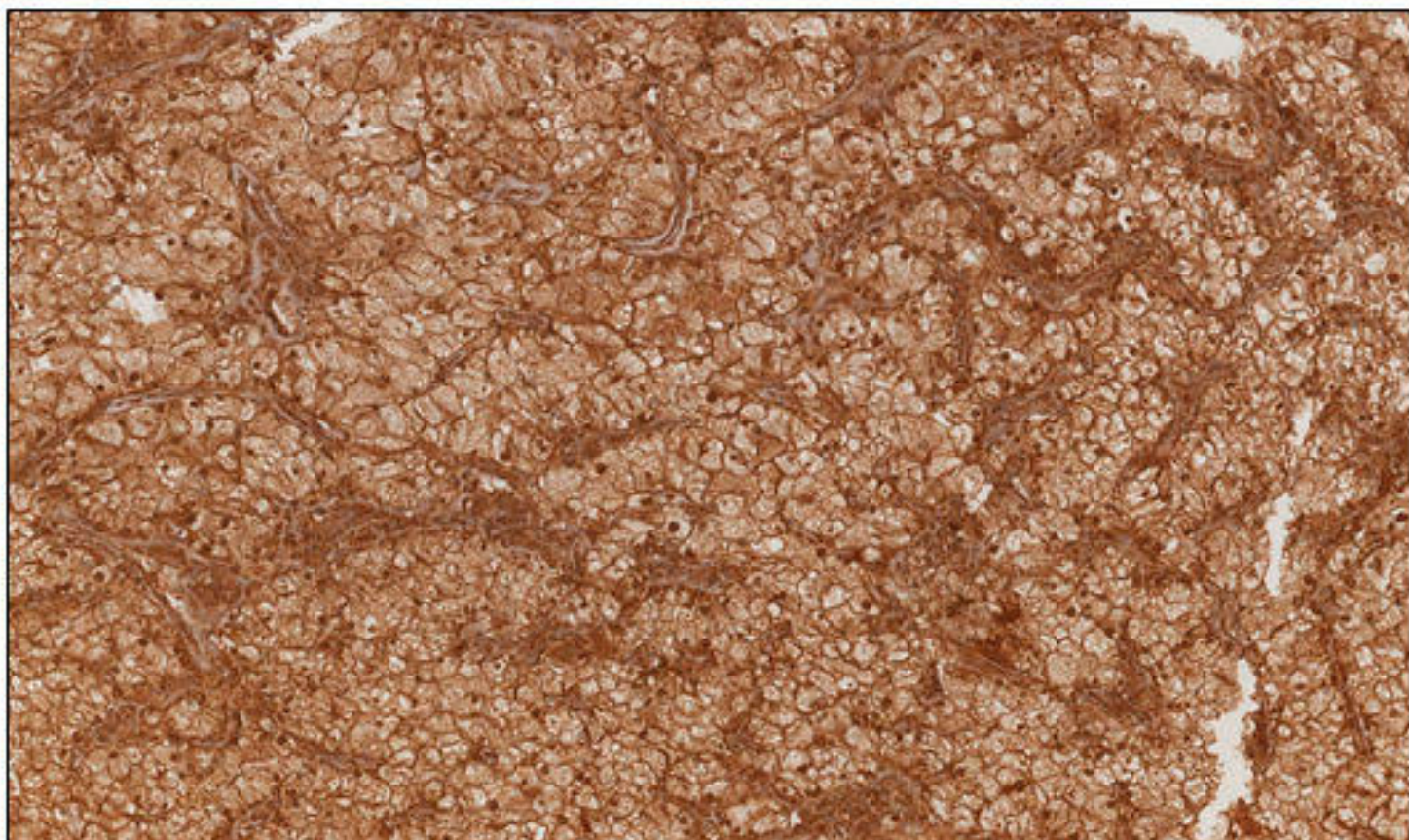

Supplement: Supplementary file 1 [file ijms-23-13968-s001.zip › Supplementary Figure S1.pdf]

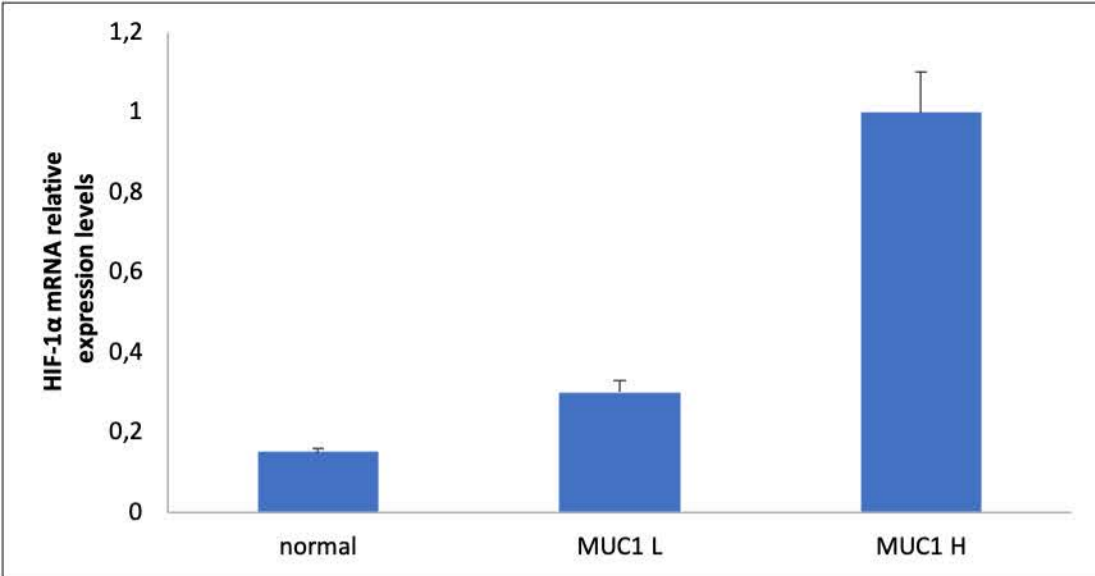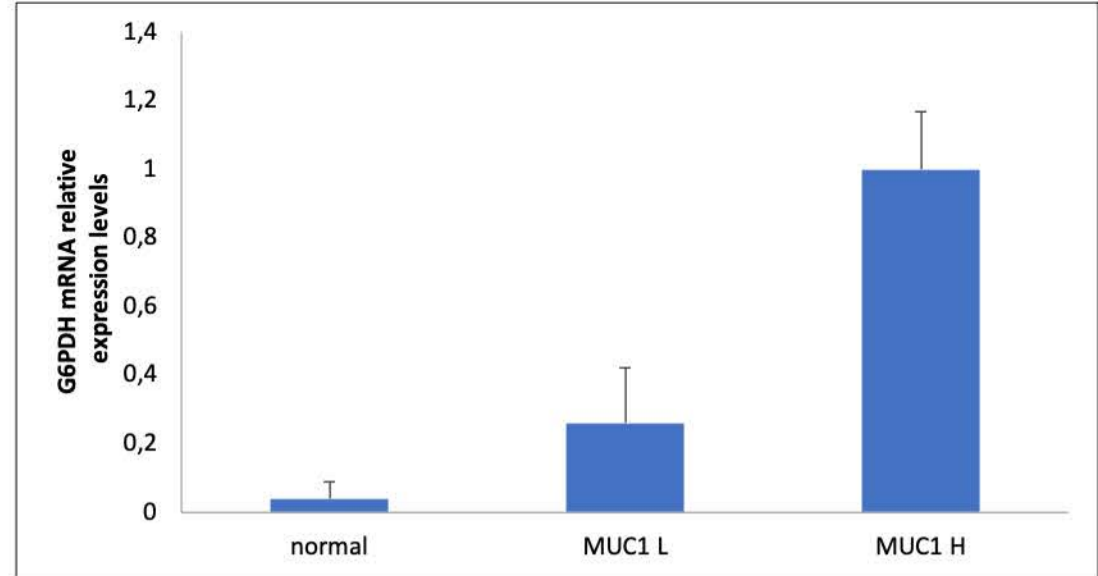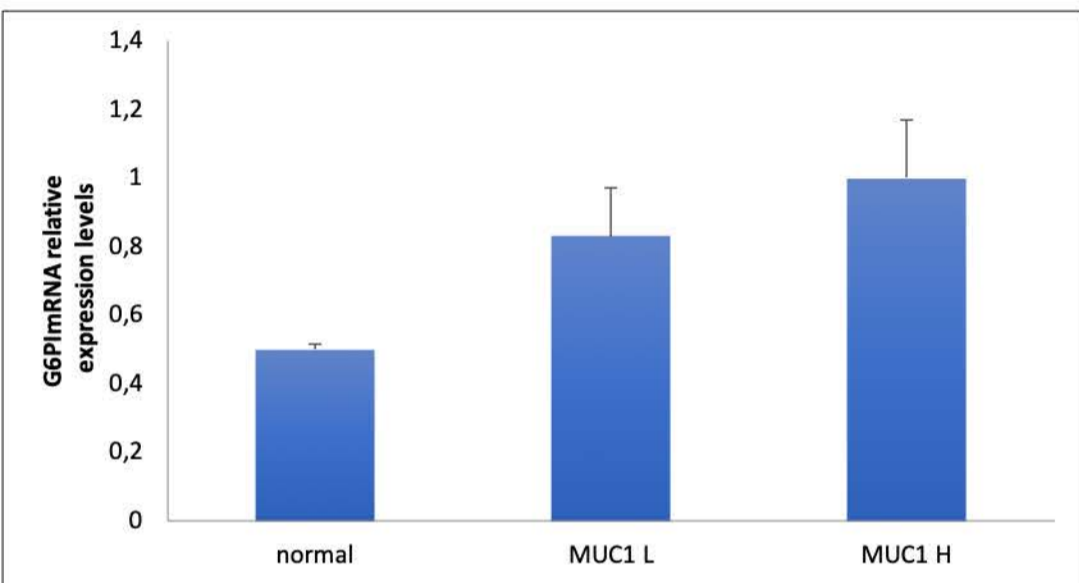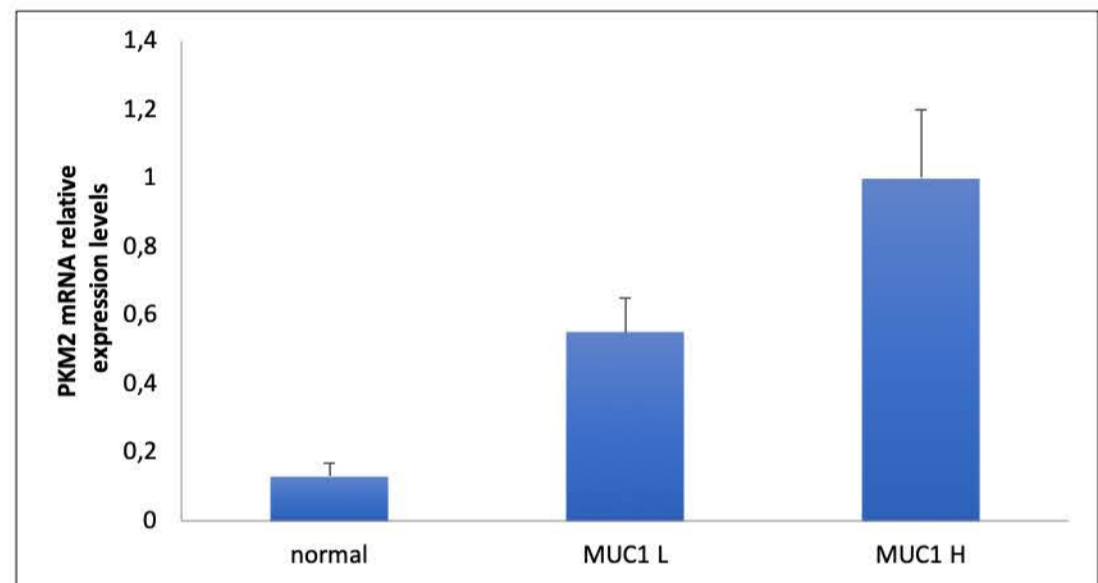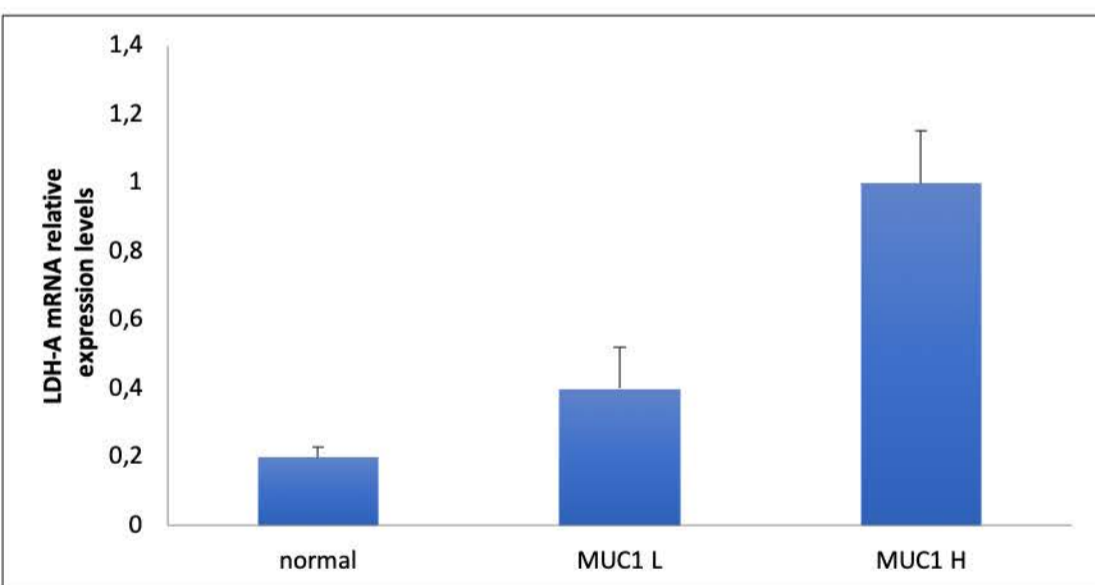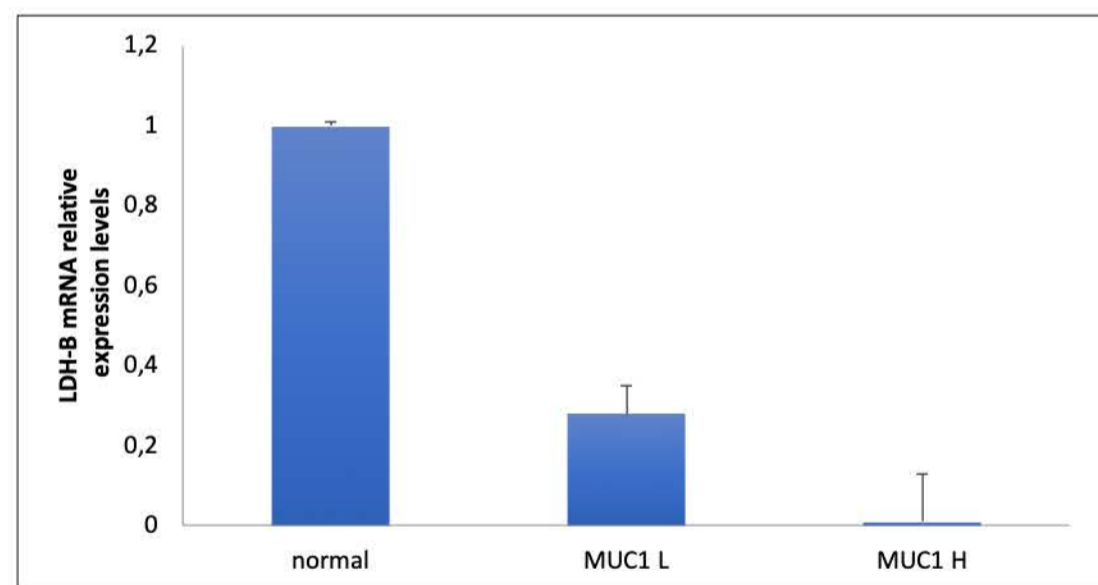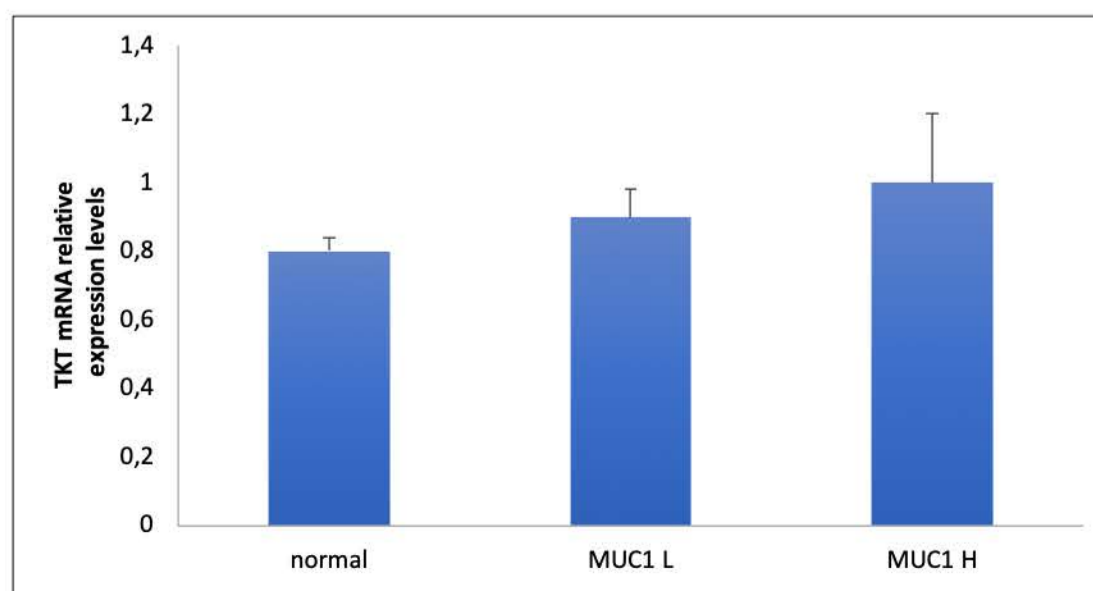

Supplement: Supplementary file 1 [file ijms-23-13968-s001.zip › Supplementary Figure S2.pdf]
